# Methylation of a CGATA element inhibits binding and regulation by GATA-1

**DOI:** 10.1038/s41467-020-16388-1

**Published:** 2020-05-22

**Authors:** Lu Yang, Zhiliang Chen, Elizabeth S. Stout, Fabien Delerue, Lars M. Ittner, Marc R. Wilkins, Kate G. R. Quinlan, Merlin Crossley

**Affiliations:** 1 School of Biotechnology and Biomolecular Sciences, UNSW Sydney, Sydney, NSW 2052 Australia; 2 Dementia Research Centre and Department of Biomedical Sciences, Faculty of Medicine and Health Sciences, Macquarie University, Sydney, NSW 2109 Australia; 3 Mark Wainwright Analytical Centre, Transgenic Animal Unit, UNSW Sydney, Sydney, NSW 2052 Australia

**Keywords:** Gene regulation, DNA methylation

## Abstract

Alterations in DNA methylation occur during development, but the mechanisms by which they influence gene expression remain uncertain. There are few examples where modification of a single CpG dinucleotide directly affects transcription factor binding and regulation of a target gene in vivo. Here, we show that the erythroid transcription factor GATA-1 — that typically binds T/AGATA sites — can also recognise CGATA elements, but only if the CpG dinucleotide is unmethylated. We focus on a single CGATA site in the *c-Kit* gene which progressively becomes unmethylated during haematopoiesis. We observe that methylation attenuates GATA-1 binding and gene regulation in cell lines. In mice, converting the CGATA element to a TGATA site that cannot be methylated leads to accumulation of megakaryocyte-erythroid progenitors. Thus, the CpG dinucleotide is essential for normal erythropoiesis and this study illustrates how a single methylated CpG can directly affect transcription factor binding and cellular regulation.

## Abstract (web summary)

While DNA methylation is thought to play a regulatory role, there are few examples where modification of a single CpG dinucleotide directly affects transcription factor binding. Here the authors show that methylation of a single CGATA element within the *c-Kit* gene inhibits binding and regulation by erythroid transcription factor GATA-1, both in cells and in mice, suggesting that methylation at this site plays an essential role in erythropoiesis.

## Introduction

DNA methylation is an important epigenetic modification that is essential to mammalian development^1^. Co-ordinated changes in DNA methylation have been documented during cellular differentiation, for example, in haematopoiesis, where many gene promoters undergo demethylation as early progenitors differentiate into granulocyte-macrophage progenitors, and genome-wide demethylation occurs during the terminal stages of erythropoiesis^2–4^. However, the mechanisms by which the DNA methylation influences gene expression and differentiation are still not fully understood.

There are numerous examples where DNA methylation at multiple CpGs, for example in CpG islands in promoters and enhancers, is accompanied by a loss of transcription factor binding^5–9^. For example, NFR1 is a DNA methylation-sensitive transcription factor whose binding is abrogated when a broad region encompassing its recognition motif is hypermethylated^8,9^.

In this study, we explored how DNA methylation at a single CpG dinucleotide could interfere with binding and regulation by GATA-1^10–13^, a critical transcription factor that modulates the expression of most if not all erythroid-specific genes^14–16^. We show that methylation of a CGATA element reduces GATA-1 binding and gene regulation in cell lines. We extend these observations by showing that a single point mutation that converts the CGATA element to a TGATA site in a regulatory region of *c-Kit*—which can still be bound by GATA-1 but that is no longer sensitive to methylation—interferes with normal haematopoiesis in mice.

## Results

### GATA-1 binds to CGATA motifs and is blocked by methylation

**Fig. 1:**
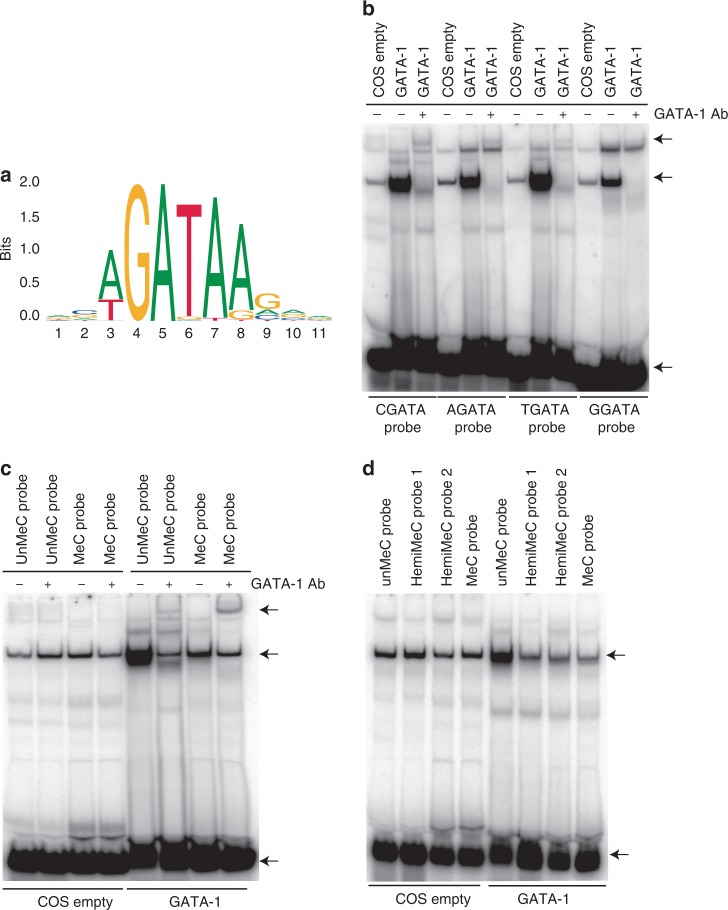
DNA methylation inhibits the binding of GATA-1 in vitro. **a** GATA-1 canonical consensus motif from JASPAR^19^. Electrophoretic mobility shift assays (EMSAs) assessing the ability of full-length mouse GATA-1 overexpressed in COS cells to bind to probes containing CGATA, AGATA, TGATA and CGATA motifs **b**, unmethylated/methylated CGATA probes **c** or unmethylated/hemi-methylated CGATA probes **d**. Untransfected COS cells are shown as a control in each case. In **b**, **c** a GATA-1 antibody has been used to super-shift the GATA-1-probe complex. The gel shifts shown in **b**–**d** were repeated three times independently with similar results. Arrows indicate migration of super-shifted GATA1, GATA-1-DNA complexes and free probes respectively. Source data are provided as a Source data file.

The previously defined GATA-1 recognition motif contains either AGATA or TGATA within a A/TGATAA/G consensus sequence (Fig. 1a)^17–19^. However, in vitro GATA-1 can also bind CGATA elements^20^. Using an electrophoretic mobility shift assay (EMSA) we confirmed that COS cell overexpressed and MEL cell endogenous GATA-1 is able to bind to CGATA, AGATA and TGATA, but less well to GGATA motifs, as previously reported^20^ (Fig. 1b and Supplementary Fig. 1a). We then focussed on the CGATA element, since this motif contains a CpG element that may be subject to methylation. Chemically synthesised probes containing unmethylated, methylated or hemi-methylated CGATA motifs were tested in EMSAs (Fig. 1c, d). This revealed that both full methylation and hemi-methylation of the CGATA motif inhibits GATA-1 binding. Probe cold competition assays support the hypothesis that GATA-1 preferentially binds to unmethylated CGATA probes (Supplementary Fig. 1b, c). In comparison with GATA-1, the related protein GATA-2 binds only weakly to probes containing CGATA motifs but this binding is also reduced by DNA methylation (Supplementary Fig. 2).

### Methylation levels change at GATA-1 bound CGATA sites

**Fig. 2:**
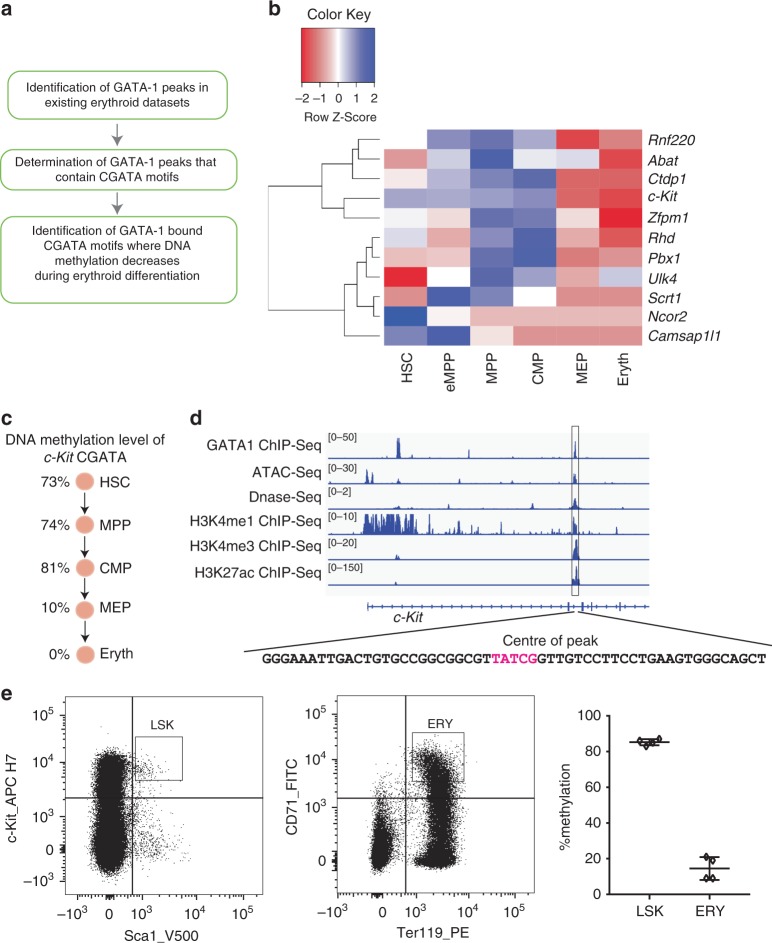
Genome-wide analysis to identify genes bound by GATA-1 with CGATA motifs where there is a change of DNA methylation status. **a** Bioinformatics analysis pipeline used to identify CGATA sites bound by GATA-1 where methylation decreases during mouse haematopoiesis^4,22^. **b** Heat-map of DNA methylation levels in blood differentiation at GATA-1 targets containing CGATA sites^22^. Genome-wide DNA methylation level has been investigated in hematopoietic stem cells (HSC), multipotent progenitor 1 (eMPP, Flk2 negative), multipotent progenitor 2 (MPP, Flk2 positive), common myeloid progenitor (CMP), megakaryocyte-erythroid progenitor (MEP) and nucleated erythroblasts (Eryth). All cells were purified from the bone marrow of adult C57BL/6 J mice^22^. **c** Schematic of mouse erythropoiesis showing changes in DNA methylation at the *c-Kit* CGATA^22^. **d** Chromatin status at CGATA site within Intron 2 of the mouse *c-Kit* gene. IGV peak tracks at CGATA (TATCG reverse complement) in GATA-1 ChIP-Seq (G1E cells)^21^, ATAC-Seq (megakaryocyte-erythroid progenitor cell)^21^, DNase-Seq (MEL cells)^31^, H3K4me1 ChIP-Seq (megakaryocyte embryo 14.5)^21^, H3K4me3 ChIP-Seq (MEL cells)^21^ and H3K27ac ChIP-Seq (MEL cells)^21^. The numbers in square brackets on the left side represent peak height. **e** Flow cytometry cell sorting was used to purify Lineage negative, Scal positive and c-Kit positive (LSK) cells and Ter119 positive and CD71 positive (erythroid; ERY) cells from mouse bone marrow. DNA methylation level at the *c-Kit* CGATA site in LSK and ERY cells was determined by pyrosequencing, *n* = 4 biologically independent animals, mean ± standard deviation. Measurements were taken from distinct samples (animals). Source data are provided as a Source data file.

We interrogated existing GATA-1 ChIP-seq data from G1E-ER4, murine erythroleukemia (MEL) cells and erythroblasts^21^ (Fig. 2a). This identified 38799 AGATA motifs, 24126 TGATA motifs and 2708 CGATA motifs that lay near the centre of GATA-1 ChIP-seq peaks reflecting in vivo binding (Supplementary Figure 3). Among all CGATA sites, there were 1139 CGATA sites which co-occurred in the same peak with (A/T)GATA motifs. We then integrated these data with genome-wide bisulphite sequencing information^4,22^ to identify individual CGATA sites where methylation levels changed during blood cell differentiation (Fig. 2b, Supplementary Figure 4 and Supplementary Table 1). Among these genes, *c-Kit* was repressed by GATA-1 while *Zfpm1* and *Rhd* were activated by GATA-1 (Supplementary Figure 5). The remaining genes, *Rnf220, Abat, Ctdp1, Pbx1, Ulk4, Scrt1, Ncor2* and *Camsap111*, did not show evidence of being regulated by GATA-1 in this system (Supplementary Figure 5). We focussed our attention on a single CGATA element within an enhancer/silencer in intron 2 of the *c-Kit* gene, a gene that encodes an important cell surface receptor for the haematopoietic growth factor, stem cell factor.

### c-Kit is a GATA-1 target gene with a regulatory CGATA site

*c-Kit* is broadly expressed in hematopoietic stem cells and progenitors, and its expression is downregulated as cell differentiation proceeds^23–25^. High expression of *c-Kit* in haematopoietic stem cells and progenitors is essential for their self-renewal and proliferation^26–28^, and the ultimate repression of *c-Kit* in the erythroid lineage is mediated in part via GATA-1^29,30^ and is associated with terminal differentiation. Existing data suggest that DNA methylation of the CGATA motif in intron 2 of *c-Kit* is high in stem cells but declines as differentiation proceeds^4,22^ (Fig. 2c, Supplementary Table 2), potentially allowing binding and repression by GATA-1. Importantly, we noted that the intron 2 CGATA element resides in a small region that in erythroid and related cells is not only notable for its strong GATA-1 ChIP-Seq peak, but is also accessible to ATAC sequencing and DNase-I mapping, and carries histone marks consistent with it being part of an functional distal regulatory element (e.g. an enhancer and/or silencer) (Fig. 2d)^21,31^.

We first compared the levels of methylation at this element, in purified murine haematopoietic stem cells and cells that had differentiated down the erythroid lineage, to assess whether methylation declined as expected. Haematopoietic stem cells (LSK; Lineage^−^, Scal^+^, c-Kit^+^) and erythroblasts (ERY, Ter119^+^, CD71^+^) were collected through flow cytometry cell sorting and subjected to pyrosequencing (Fig. 2e). Consistent with previous genome-wide bisulphite sequencing data, the site is highly methylated in LSKs (~80%) but hypomethylated in the erythroid lineage (~10%) (Fig. 2e).

We next moved to cellular models to investigate whether GATA-1 could bind and regulate *c-Kit* expression from this element in living cells, and whether mutation of this motif, or alteration in its methylation status affected GATA-1 binding and *c-Kit* regulation. It should be noted that while GATA-1 can serve as an activator or repressor, it is known to repress *c-Kit* in maturing erythroid cells through multiple elements^29,30^. Therefore, we anticipated that disrupting the CGATA motif in intron 2 might de-repress *c-Kit* in erythroid cell lines.

**Fig. 3:**
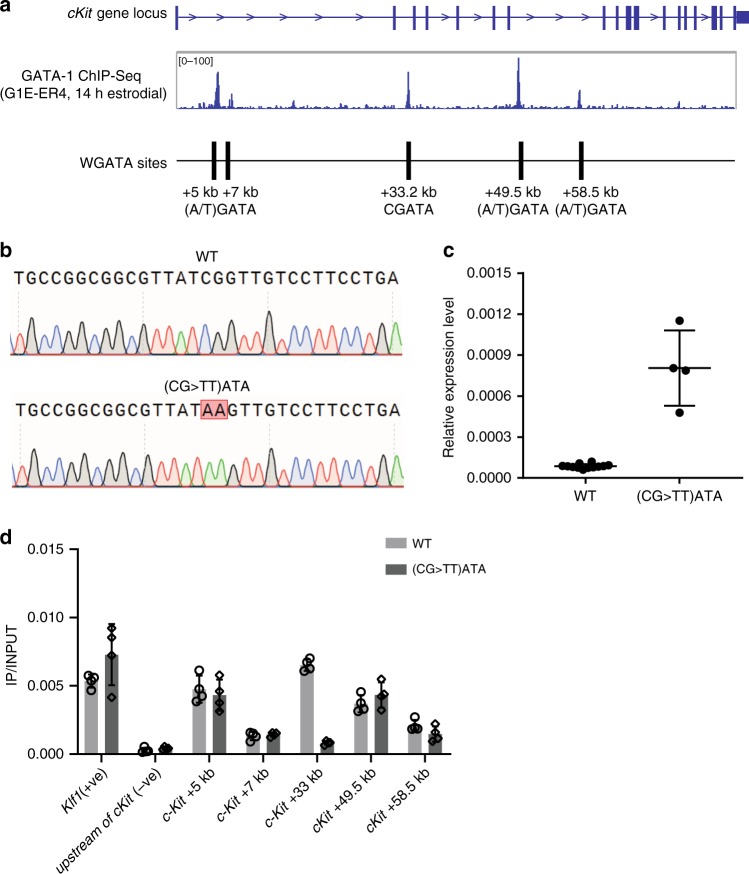
The CGATA site at c-Kit intron 2 is a regulatory site bound by GATA-1 in MEL cells. **a** Schematic of GATA sites in the *c-Kit* gene locus^21^. **b** Sequencing confirms that a (CG > TT)ATA mutation was introduced at *c-Kit* intron 2. **c** The mRNA expression level of *c-Kit* in wild type and (CG > TT)ATA mutant clones, *n* = 11 biologically independent cells for WT, *n* = 4 biologically independent cells for mutant, mean ± standard deviation. Measurements were taken from distinct samples (separate clonal cell populations). **d** GATA-1 ChIP qPCR in wild type and mutant (CG > TT)ATA clones, n = 4 biologically independent cells, mean ± standard deviation. The promoter region of *Klf1* served as a positive control (+ve). An upstream region distal to the *c-Kit* gene was used as a negative control (−ve). All GATA consensus binding sites with evidence of GATA-1 binding in ChIP-seq studies in the *c-Kit* gene locus have been tested (+5, +7, +33, +49.5, +58 kb; see **a**). All experiments were performed in undifferentiated MEL cells. Source data are provided as a Source data file.

To test whether the CGATA motif in *c-Kit* intron 2 is regulatory, we utilised MEL cells in which the CGATA element is essentially unmethylated (Supplementary Figure 6) and in which we therefore expected to observe GATA-1 binding (Fig. 3a). ChIP assays confirmed that GATA-1 binds this site in MEL cells (Fig. 3d). We then used CRISPR-mediated editing in MEL cells to mutate the *c-Kit* +33 kb CGATA element to TTATA to disrupt the motif (Fig. 3b) and tested whether this prevented GATA-1 binding. As expected, this mutation disrupted GATA-1 binding without affecting GATA-1 binding to the positive control site or at other bound GATA sites within the *c-Kit* gene locus (Fig. 3d). We also tested the impact of this mutation on *c-Kit* expression and saw a clear de-repression (Fig. 3c), consistent with the view that GATA-1 can bind and repress *c-Kit* at least in part via binding the intron 2 CGATA element that is unmethylated and able to be bound in mature erythroid cells.

### Increasing c-Kit CGATA site methylation alters regulation

**Fig. 4:**
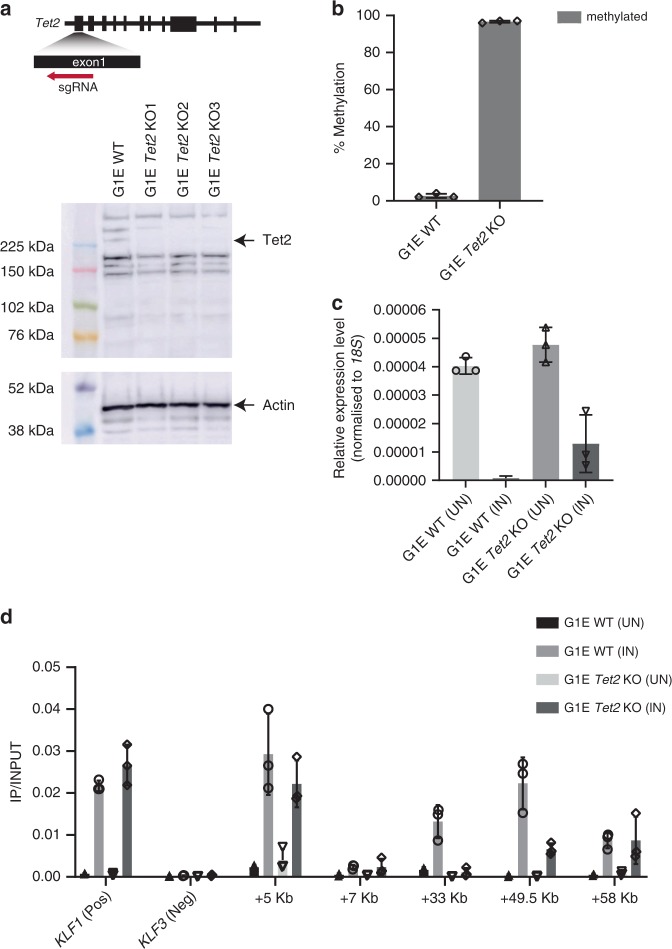
DNA methylation blocks the binding of GATA-1 to the c-Kit CGATA site and attenuates repression in G1E-ER4 cells. **a** CRISPR/Cas9 was used to knock out *Tet2* in G1E-ER4 cells. Western blot using an antibody to Tet2 identifies clones where *Tet2* has been knocked out. A blot using an antibody to actin is shown as a loading control. Three *Tet2* knock out clones and clonal wild-type controls were selected for further investigation. The experiment was repeated 3 times independently with similar results. **b** Methylation level by pyrosequencing of the *c-Kit* Intron 2 CGATA site in three clonal G1E WT and G1E *Tet2* knock out cells (n = 3 biologically independent cells), mean ± standard deviation. **c** The expression level of *c-Kit* in G1E WT and G1E *Tet2* KO cells by qPCR. Expressed relative to 18S expression, *n* = 3 biologically independent cells, mean ± standard deviation. Cells were induced with the addition of tamoxifen for 24 h to restore GATA-1-ER fusion proteins in cell nucleus (TAM). Cells incubated with ethanol were used as mock control (NC). **d** GATA-1 ChIP-qPCR in wild type and G1E *Tet2* cells after 24 h of GATA-1 induction by incubating with tamoxifen. *KLF1* promoter region was used a positive control (+ve). An upstream region distal to the *KLF3* gene was used as a negative control (−ve), the CGATA site at c-Kit intron 2 is the +33 site, *n* = 3 biologically independent cells, mean ± standard deviation, ChIP-qPCR was repeated two times independently with similar results. Source data are provided as a Source data file.

To investigate if DNA methylation of the CGATA element could block GATA-1 binding and whether it influenced the functional repression of *c-Kit* by GATA-1, we employed another murine erythroid cell line, G1E-ER4. This line has become a useful model for functional studies as it expresses an estrogen inducible GATA-1-ER fusion^13,32–34^. We tested for methylation of the CGATA element in G1E-ER4 cells and found the site was largely unmethylated (Fig. 4b, Supplementary Fig. 8). Reasoning that transient methylation might be being erased by demethylases of the Tet family^35^, we checked the expression of *Tet1, Tet2 and Tet3*. We found that *Tet2* was highly expressed (Supplementary Fig. 7) and used CRISPR-mediated gene editing to knock out *Tet2* in G1E-ER4 cells (Fig. 4a). This disruption was associated with ensuing methylation of the CGATA element (Fig. 4b, Supplementary Fig. 9). GATA-1 is inducible (Supplementary Fig. 10a) and leads to G1E-ER4 cell differentiation (Supplementary Fig. 10b) in both wild type and *Tet2* knock out G1E-ER4 cells. *Tet2* knock out did not influence G1E-ER4 cell survival or proliferation (Supplementary Fig. 10c, d).

We tested the binding of GATA-1 to the CGATA element using ChIP assays in wildtype and *Tet2* knockout cells and as expected found reduced GATA-1 binding to the CGATA +33 kb site in the *c-Kit* promoter in knockout cells where methylation levels are higher (Fig. 4d). Interestingly, GATA-1 binding was also reduced at intron 4 of the *c-Kit* gene (49.5 kb from the TSS), suggesting the that binding to this site might be also influenced by DNA methylation possibly indirectly via chromatin conformation or some other mechanism since this is not a CGATA element (Fig. 4d). However, global hypermethylation did not affect the binding of GATA-1 at a positive control region or other GATA sites in *c-Kit* gene locus (Fig. 4d). This in vivo result is consistent with the in vitro EMSA assays (Fig. 1), suggesting that methylation at CGATA sites blocks GATA-1 binding. We then considered function. Importantly, the overall levels of *c-Kit* were comparable in the wildtype and the *Tet2* knockout lines before GATA-1 induction with tamoxifen, suggesting that no major perturbation of the chromatin configuration at the locus had occurred (Fig. 4c). When GATA-1 is induced in the wildtype line *c-Kit* expression diminishes, consistent with previous results^30^. In *Tet2* knockout cells, where the CGATA site is more heavily methylated, GATA-1-mediated repression was attenuated (Fig. 4c). In summary, our results confirm that methylation modulates GATA-1 binding in cell lines and suggest that *c-Kit* regulation is also attenuated in vivo.

### Methylation at the c-Kit CGATA site is important in mice

**Fig. 5:**
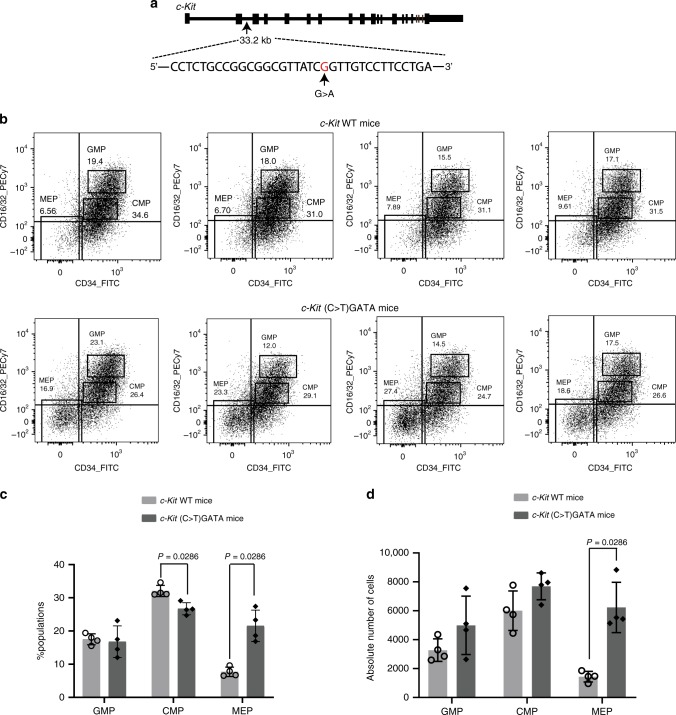
Introduction of a single C > T mutation in the CGATA site in c-Kit intron 2 in a mouse model demonstrates that the CpG at this site plays a role in haematopoiesis. **a** A (C > T)GATA mutation was introduced at *c-Kit* Intron 2 in C57BL/6J mice using CRISPR/Cas9. **b** Flow cytometry analysis of progenitor populations in mouse bone marrow from WT and homozygous (C > T)GATA mutant mouse littermates. CD16/32 low and CD34 negative cells are megakaryocyte-erythroid progenitors (MEPs). CD16/32 medium and CD34 positive cells are common-myeloid progenitors (CMPs). CD16/32 high and CD34 positive cells are granulocyte-monocyte progenitors (GMPs). All progenitors were derived from lineage negative, Scal negative, c-Kit positive (LSK) populations. **c**, **d** The percentage and absolute cell number of different populations in each mouse have been summarised in the histogram, *n* = 4 biologically independent animals, mean ± standard deviation. Mann–Whitney U, two-tailed statistical test, *P-*values shown. Measurements were taken from distinct samples (animals). Source data are provided as a Source data file.

To definitively determine whether this single CpG dinucleotide is required for *c-Kit* regulation during haematopoiesis in vivo we tested the importance of this site using CRISPR gene editing in mice. Rather than simply disrupting the site, we introduced a C > T mutation, to convert the CGATA element to a TGATA site (Fig. 5a). We reasoned that this site would now be bound constitutively, rather than only after the onset of de-methylation during haematopoiesis. Bone marrow samples were collected from wild type and homozygous mutant mice (Supplementary Fig. 11a). The overt phenotype of the mutant mice and blood counts were not significantly different from wildtype (Supplementary Fig. 11b). We investigated the proportion and absolute number of bone marrow precursors and proportion of erythroid populations using flow cytometry. There was no change in the proportion of hematopoietic stem cells, multipotent progenitor populations or erythroid populations between wild type mice and mutant mice (Supplementary Fig. 12). However, we detected a clear accumulation of megakaryocyte-erythroid progenitors (MEPs) in mutant mice (Fig. 5b). In addition, in mutant mice, the overall expression level of *c-Kit* in MEPs was significantly reduced in comparison to that of wild type mice (Supplementary Fig. 13). This result is consistent with this single CpG dinucleotide playing a role in haematopoietic differentiation in vivo.

## Discussion

In vivo GATA-1 has been implicated in binding primarily to AGATA or TGATA elements, and not to CGATA sites, but this may be a consequence of genomic ChIP-Seq analyses under-appreciating the importance of CGATA sites, due to the rarity of CpG dinucleotides in mammalian genomes. In addition, since CpG dinucleotides are often methylated the subset of CGATA elements bound by GATA-1 will be further limited. Nevertheless, the fact that CGATA elements can be subjected to methylation, and that this influences binding of GATA-1, means that such elements may be instrumental in mediating the effects of alterations in methylation that have been observed to occur during haematopoietic differentiation.

**Fig. 6:**
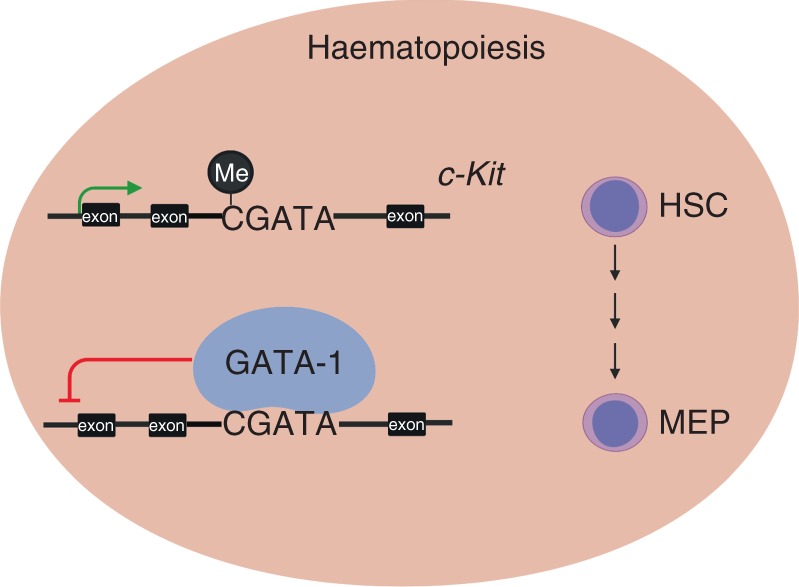
Model to show role of DNA methylation and impact of forced demethylation at the CGATA site in c-Kit intron 2 on haematopoiesis. In wild type mice, the CGATA site at intron 2 of *c-Kit* gene is highly methylated at early stages of haematopoiesis and gradually becomes de-methylated during haematopoiesis. GATA-1 binds to the CGATA site at intron 2 of the *c-Kit* gene when it is de-methylated and represses the expression of *c-Kit*. In mutant mice, the (C > T)GATA mutation is unable to be modified by DNA methylation and GATA-1 can bind to this site at earlier stages of haematopoiesis. We observe a decrease in c-Kit expression and an increase in MEP cell number in mice homozygous for this *c-Kit* (C > T)GATA mutation. Note that the diagram illustrates that methylation of the CGATA element is required for normal regulation of *c-Kit* but is not likely to be sufficient for the full regulation of the *c-Kit* gene. Other GATA elements in additional regulatory elements are also likely to be relevant but for brevity are not included here.

Our work provides a clear illustration that at least one CpG dinucleotide within a CGATA site, that is progressively demethylated during haematopoiesis, is involved in *c-Kit* regulation in vivo. Methylation of the site inhibits GATA-1 binding in vitro and in cellular assays, and modestly impairs repression by GATA-1. A single point mutation that converts the CGATA element to a TGATA site—which can still be bound by GATA-1 but that can no longer provide a methylation sensitive binding site—interferes with normal haematopoiesis in mice (Fig. 6). These results suggest that DNA methylation at the binding site inhibits GATA-1 binding and show that a single residue’s ability to be methylated can influence gene regulation in vivo.

## Methods

### EMSA

EMSA were performed^36^. The sense oligonucleotide was labelled with [γ-^32^P] triphosphate before annealing with the antisense strand via slow cooling from 100 °C to room temperature. Labelled duplex DNA oligonucleotides were purified with Quick Spin Columns (Sigma-Aldrich, #11273922001). In all, 5 μg pMT3-GATA-1 (full length) was transfected into COS-7 cells (gift from Stu Orkin, Harvard Medical School Boston, USA) for 48 h using Fugene6 (Promega, #E2691). Nuclear extracts were harvested from COS-7 cells^37^. Nuclear extracts were incubated with radiolabelled probes at 4 °C for 10 min. The protein-probe mixtures were run at 4 °C on a 6% native polyacrylamide gel in TBE buffer (45 mM Tris, 45 mM boric acid, 1 mM EDTA). COS empty lane was included as control to show any background binding to the probe. Super-shifting of GATA-1 was achieved using an anti-GATA-1 antibody (Santa Cruz technology, #sc-256, 1:5000 dilution). Gels were run for 1 h and 30 min, at 250 volts. After running, gels were dried under vacuum. Dried gels were exposed to a FULJIFILM BAS CASSETTE2 2025 phosphor screen overnight followed by visualised using a FUJIFILM FLA-5100 Fluorescent Image Analyser. All probes used in EMSAs are listed in Supplementary Table 3.

### Western blot

Nuclear extracts from cultured G1E-ER4 cells (gift from Stu Orkin, Harvard Medical School Boston, USA) were made^37^. To detect Tet2 expression, 60 μg of cell nuclear extract was loaded onto a NuPAGE^TM^ Novex^TM^ 10% Bis-Tris Protein Gel (ThermoFisher Scientific, #NP0301BOX) and gels were run in MOPS buffer at 200 V for 60 min. Proteins were transferred onto a nitrocellulose membrane at 30 V for 70 min. Blots were blocked with 4% skim milk for 30 min. After blocking, blots were probed for Tet2 and Actin using an anti-Tet2 antibody (Abcam, #ab124297, 1:5000 dilution) and an anti-β-Actin antibody (Sigma, #A1978, 1:10000 dilution) respectively.

### RNA extraction and cDNA synthesis

Total RNA was extracted with Tri-reagent according to the manufacturer’s protocol (Qiagen, #74004). RNA was treated with a DNA removal kit (Qiagen, #79254) to ensure the purity of RNA samples. RNA concentrations were determined by UV-light absorbance at 260 nm using a Nanodrop (ThermoFisher Scientific).

Approximately 500 ng of RNA was used for cDNA synthesis with SuperScript VILO cDNA synthesis kit (ThermoFisher Scientific, Cat#11755050) in which random hexamers were used as primers for generation of first strand cDNA. For each sample, a negative control was also set up in the absence of SuperScript III reverse transcriptase (RT) to check for genomic DNA contamination.

### Quantitative real-time PCR

Quantitative real-time PCRs (qPCR) were set up with SYBR Green PCR Master Mix, forward and reverse primers targeting locus of interest and template of cDNA or ChIP DNA. All reactions were set up in duplicate and run on the Applied Biosystems ViiA7 Real-Time PCR system (Applied Biosystems). For transcript level analysis, the results were normalised to the 18S rRNA levels of the respective samples. For ChIP experiments, the results were normalised against the respective input samples containing total sonicated genomic DNA. Primers used for qPCR are listed in Supplementary Table 4.

### Cell culture (G1E-ER4, MEL)

The murine erythroleukaemia cell line (MEL, gift from Stu Orkin, Harvard Medical School Boston, USA) was cultured in Dulbecco’s Modified Eagle Medium (DMEM) (Life Technologies, #435847) supplemented with 10% (v/v) foetal calf serum (FCS) (Interpath, #SFBSF13) and 1% (v/v) penicillin/streptomycin/glutamine (PSG) solution (Life Technologies, #10378016)^38^. G1E-ER4 is a subclone of GATA-1 null erythroblasts which stably expresses a GATA-1-ER (estradiol receptor ligand binding domain) fusion protein^32^. G1E-ER4 cells were cultured in Iscove’s MDM medium (Life Technologies, #12440-061) supplemented with 15% heat-inactivated foetal calf serum (Interpath, #SFBSF13), 1% PSG (Life Technologies, #10378016), 62 μl of 10% monothioglycerol (Sigma-Aldrich, #M6145), 0.6% home-made Kit-ligand conditioned medium, 50ul mouse erythropoietin (Biolegend, #587606) in 500 ml IMDM medium^39^. Kit-ligand conditioned medium was made from CHO cells (gift from Stu Orkin, Harvard Medical School Boston, USA)^32^. Exponentially proliferating G1E-ER4 cells were induced with the addition of 4-hydroxytamoxifen (tamoxifen) (Sigma, #H7904) to a final concentration of 0.1 nmol for 24 h to restore GATA-1-ER fusion protein into cell nucleus^32^. G1E-ER4 cells incubated with ethanol (1 ul ethanol in 10 ml media) was used as mock (NC) control.

### Chromatin immunoprecipitation

Chromatin immunoprecipitation (ChIP) experiments were performed^40^. In all, 7 × 10^7^−1 × 10^8^ cells were used for each ChIP. Cells were crosslinked with 1% formaldehyde for 10 min and the reaction was quenched by addition of 2.5 mM glycine. Fixed cells were sonicated at high voltage for 20 min (30 s on, 30 seconds off) using Bioruptor® (Diagenode) to obtain 200–300 bp DNA fragments. The fragmented chromatin was pulled down at 4 °C overnight using an antibody against GATA-1 (Santa Cruz Biotechnology, #sc-256, 1:5000 dilution). Chromatin cross-linking was then reversed at 65 °C overnight followed by DNA purification. Real-time qPCR was performed on ChIP DNA on an Applied Biosystems ViiA7 Real-time PCR System (Applied Biosystems). Primers used for ChIP-qPCR are listed in Supplementary Table 5.

### Pyrosequencing

Genomic DNA was extracted and purified from cells using a PureLink Genomic DNA Mini Kit, according to the manufacturer’s instructions (Life Technologies, #K1820-02). Briefly, harvested cells were lysed at 55 °C for 10 min and then gDNA was precipitated by adding 100% ethanol and purified through the provided column. Genomic DNA was treated with bisulphite and purified following using a EpiTect Fast Bisulfite Conversion Kit, following manufacturer’s instructions (Qiagen, #59824). Target regions were amplified from bisulphite converted gDNA using MyTaq HS DNA polymerase (Bioline, #BIO-25045) with Biotin-labelled primers. Pyrosequencing of samples were performed by Australia Genome Research Facility (AGRF) using the PyroMark Q24 platform. All primers used for pyrosequencing are listed in Supplementary Table 6.

### CRISPR/Cas9 genome editing in cell lines

Single guide RNAs (sgRNAs) were designed using the website [https://www.benchling.com/crispr/] and guides with low off-target scores were selected^41^. sgRNAs were cloned into pSpCa9(BB)−2A-GFP (px458) plasmid (Addgene, #48138)^41^. *Tet2* knock out cells were generated by CRISPR/Cas9 using non-homologous end joining (NHEJ). For site specific mutations, a DNA donor template was used to drive homology-directed repair (HDR). In all, 150 bp DNA donor templates for genomic modification were synthesised by Integrated DNA Technologies (IDT), containing the mutation in the middle of the sequence. sgRNA plasmid and DNA donor were co-transfected into cells using Neon transfection system (ThermoFisher). In all, 48 h after transfection, cells were sorted with BD FACSAriaIII flow cytometer for those positive for GFP (indicating that they had taken up the pSpCa9(BB)−2A-GFP plasmid) and negative for the live-dead marker 7-AAD (BIO-RAD, #1351102, one drop per sample). After a further 48 h, GFP and 7-AAD negative cells were single sorted into 96-well plates. After a week, clonal populations derived from single cells were screened through Sanger sequencing and western blot. Primers and donors used for genome editing are listed in Supplementary Table 7.

### BrdU cell proliferation assay

The proliferation of G1E-ER4 cells was assessed by using a BrdU cell proliferation assay kit, according to the manufacturer’s instructions (Cell Signaling Technology, #6813). We plated 1 × 10^4^ G1E-ER4 cells into wells of a 96-well plate, added 10 μl 1x BrdU into each well and incubated for 4, 8 or 24 h. After the incubation, the cells were fixed and denatured for 30 min, then 100 μl/well BrdU detection antibody solution (Cell Signaling Technology, #94079, 1:100 dilution) was added and incubate at room temperature for 1 h. After the incubation, each well was washed three times with 100 μl wash buffer. Next 100 μl/well HRP-conjugated secondary antibody solution (Cell Signaling Technology, #34709, 1:100 dilution) was added and incubated at room temperature for 30 min. After the incubation, the cells were washed three times with 100 μl wash buffer. TMB substrate (100 μl) was added and incubated for 30 min before adding stopping buffer. After adding the stopping buffer, absorbance was read at 450 nm to detect cell proliferation.

### Generation of c-Kit (C > T)GATA site mutant mice

Generation of mice was done with approval of the UNSW Animal Care and Ethics Committee (Approval No. 16/19A). The CRISPR/Cas9 system was utilised for c-Kit (C > T)GATA mouse generation by injecting sgRNA and Cas9 protein (EnGen^®^ Spy Cas9 NLS) into fertilised oocytes^42^. Briefly, sgRNAs were designed using the website [https://www.benchling.com/crispr/]^41^. A linearised DNA template for each sgRNA was generated using a non-cloning method by virtue of a T7-conjugated PCR. The forward primer contains the T7 promoter minimal sequence, upstream of the 20 bp sgRNA sequence and a sequence complimentary to the 5′ end of the sgRNA scaffold of pSpCa9(BB)−2A-GFP. The reverse primer is complimentary to the 3′ end of the sgRNA scaffold of pSpCa9(BB)−2A-GFP. The linearised DNA was amplified within T7 forward primer and sgRNA scaffold reverse primer using Q5 polymerase (NEB, #M0491). The resulting linearised DNA was in vitro transcribed into sgRNA using a T7 Quick High Yield RNA synthesis kit, following the manufacturer’s instructions (NEB, #E2050S). sgRNAs were purified using NucAway Spin columns (ThermoFisher, #AM10070). The donor DNA template was synthesised from Integrated DNA Technologies (IDT). The sgRNA, Cas9 protein and DNA donor were microinjected into C57BL/6J mouse oocytes at 0.5 days post-coitum (dpc) and immediately transferred to foster mothers (Swiss strain). The C57BL/6J offspring were genotyped through Sanger sequencing. Briefly, mouse tails were digested in Direct PCR (tail) lysis reagent (Australian Biosearch, #AB-102-T) at 55 °C overnight and then heat inactivated at 85 °C for 45 min. In all, 1 μl of lysates were used as templates for a genotyping PCR using Q5 polymerase (NEB, #0491). PCR products were Sanger sequenced to check for the presence of the mutation. Mice that were heterozygous for the c-Kit (C > T)GATA mutation were backcrossed with wild type C57BL/6J mice for five generations to eliminate potential off-target effects. Primers used for in vitro transcription and genotyping are listed in Supplementary Table 8.

### Animal husbandry

All animal work was carried out in accordance with approval from the UNSW Animal Care and Ethics Committee (Approval Nos. 16/5B and 18/156B). Animals were housed in a specific pathogen-free environment, at a constant ambient temperature of 22 °C, on a 12 h high-dark cycle and with ad libitum access to standard chow food and water.

### Flow cytometry analysis of erythrocyte populations

Mouse bone marrow cells were stained with CD71 (Biolegend, #334104, 1:100 dilution), Ter119 (BD Bioscience, #553673, 1:200 dilution) and DAPI (Life Technologies, #62248) for 30 min. After staining, cells were washed with 3 ml FACS buffer and centrifuged at 300×*g* at 4 °C for 5 min. Cells were resuspended in 500ul FACS buffer. Stained samples were run on BD LSRFortessa SORP flow cytometer. Data was collected using BD FACSDiva software and analysed using FlowJo V10 software. Flow cytometry gating strategies are shown in Supplementary Fig. 14b.

### Flow cytometry analysis of HSPCs

Haematopoietic stem cell and progenitor cells (HSPCs) were analysed by flow cytometry as described below. Mouse bone marrow was lysed with 9 ml RO water for 10 s followed by adding 1 ml 10x PBS to get rid of mature red blood cells since mature red blood cells do not have cell nucleus and will burst immediately under low osmotic pressure in water. Lysed bone marrow cells were stained with biotin-conjugated lineage marker antibody cocktail:CD3 monoclonal antibody (ThermoFisher Scientific, #13-0037-82, 1:200 dilution), CD4 monoclonal antibody (ThermoFisher Scientific, #13-0041-85, 1:100 dilution),CD11b monoclonal antibody (ThermoFisher Scientific, #13-0112-85, 1:100 dilution), CD5 monoclonal antibody (ThermoFisher Scientific, #13-0051-85, 1:200 dilution), CD8 monoclonal antibody (ThermoFisher Scientific, #13-0081-85, 1:200 dilution), CD45R (B220) monoclonal antibody (ThermoFisher Scientific, #13-0452-85, 1:200 dilution), Ly-6G monoclonal (ThermoFisher Scientific, #13-5931-85, 1:200 dilution),TER-119 monoclonal antibody (ThermoFisher Scientific, #13-5921-82, #1:200 dilution), at 4 °C for 30 min. Stained cells were washed with FACS buffer (1xPBS, 5% FCS, 2 mM EDTA) and centrifuged at 300 × *g* at 4 °C for 5 minutes. Cells were resuspended with FACS buffer and stained with PE-CF594 Streptavidin (BD Bioscience, #562318, 1:300 dilution),, LY-6A/E(Sca1)-V500 antibody (BD Bioscience, #561229, 1:200 dilution), CD117(cKit)-APC-H7 antibody(BD Bioscience, #560250, 1:200 dilution), CD34-FITC antibody (BD Bioscience, #553733, 1:100 dilution), CD16/32-PE-Cy7 antibody (ThermoFisher, #25-0161-81, 1:200 dilution) and DAPI (Life Technologies, #62248) at 4 °C for 30 min. After staining, cells were washed with 3 ml FACS buffer and centrifuged at 300 × *g* at 4 °C for 5 min. Cells were resuspended in 500 μl FACS buffer. Stained samples were run on BD LSRFortessa SORP flow cytometer. Data was collected using BD FACSDiva software and analysed using FlowJo V10 software. Flow cytometry gating strategies are shown in Supplementary Fig. 14a.

### Peripheral blood count

Blood was collected by cardiac puncture from wild type and *c-Kit* (C > T)GATA homozygous mutant mice and added to 50 μl 100U ml^−1^ heparin (Sigma, #H3149-250KU) in BD vacutainer blood collection tubes (BD Bioscience, #367839). Blood counts were measured by Sysmex XN-1000RF. Original numbers of cells were calculated using the following dilution factor formula:

$${\mathrm{Correct}}\,{\mathrm{number}}\,{\mathrm{of}}\,{\mathrm{cells}} = \frac{{{\mathrm{X}}\;{\mathrm{{{\mu}}l}}\,{\mathrm{of}}\,{\mathrm{blood}} + 50\;{\mathrm{{\mu}l}}}}{{{\mathrm{X}}\;{\mathrm{{{\mu}}l}}\,{\mathrm{of}}\,{\mathrm{blood}}}}\;\times\;{\mathrm{Count}}\,{\mathrm{number}}\,{\mathrm{of}}\,{\mathrm{cells}}$$

### Bioinformatic analysis

GATA-1 ChIP-Seq datasets from different cell types were download from the ENCODE portal^43^ [https://www.encodeproject.org]. Broad peaks were first split into narrow peaks using *PeakSplitter* function in *PeakAnalyzer* software^44^ for improving individual subpeaks’ analysis. Different components from *MEME SUITE* was used to perform peak and motif analysis: Comprehensive motif analysis was performed using *MEME ChIP*^45,46^; *FIMO*^45^ was then used to scan the peaks containing ‘CGATA’ motifs. Finally peak annotation was performed using *PeakAnnotator* function in *PeakAnalyzer*^44^. Reduced representation bisulphite sequencing (RRBS) datasets from blood cells and foetal liver tissues were downloaded from GEO^4,22^. Single CpG methylation data from different stages of erythroid maturation was analysed using the Bioconductor package *RnBeads*^47^. DNA methylation levels of CGATA Peaks were measured by *BEDtools*^48^ from *RnBeads* output. Heatmaps of DNA methylation changes of CGATA Peaks were drawn using gplots library^49^ in R. All dataset accession numbers are listed in Supplementary Table 9.

### Statistical analysis

The mean and standard deviation (SD) are shown for the data in each figure, except where *n* < 3. Two-tailed Mann–Whitney U (non-parametric) tests were performed to assess the significance of differences of proportions of common-myeloid progenitors (CMPs) megakaryocyte-erythroid progenitors (MEPs) in bone marrow between wild type and (C > T)GATA mutant mice. The statistical analyses for ChIP-Seq and RRBS experiments were performed by various software programs, details are provided above.

### Reporting summary

Further information on research design is available in the Nature Research Reporting Summary linked to this article.

## Supplementary information

Supplementary Information

Reporting summary

## Data Availability

The data that support the findings of this study are available from the corresponding author upon reasonable request. All ChIP-Seq, ATAC-Seq and DNase-Seq data were downloaded from Encyclopedia of DNA Elements Database (ENCODE) (Supplementary Table 9). All Reduced Representation Bisulfite Sequencing (RRBS) data were downloaded from Gene Expression Omnibus (GEO) database (Supplementary Table 9). Publicly available DNA methylation dynamics data were open sources online (http://www.medical- epigenomics.org/papers/broad_mirror/invivomethylation/index.html). The source data underlying Figs. 1b–d, 2e, 3c, d, 4a–d and 5c, d and Supplementary Figs 1a, b, 2a, b, 5, 7, 10a–d, 11b, 12a–c and 13 are provided as a Source data file.

## Source data

Source Data
