# Supplementary material for: Methylation of a CGATA element inhibits binding and regulation by GATA-1
Source: Nat Commun. 2020 May 22;11:2560. doi: 10.1038/s41467-020-16388-1 (PMC7244756; doi:10.1038/s41467-020-16388-1)
Supplement: Supplementary file 1 — Supplementary Information [file 41467_2020_16388_MOESM1_ESM.pdf]

Supplementary Information

"Methylation of a CGATA element inhibits binding and regulation by GATA-1"

Yang et al.

Nature Communications, 2020

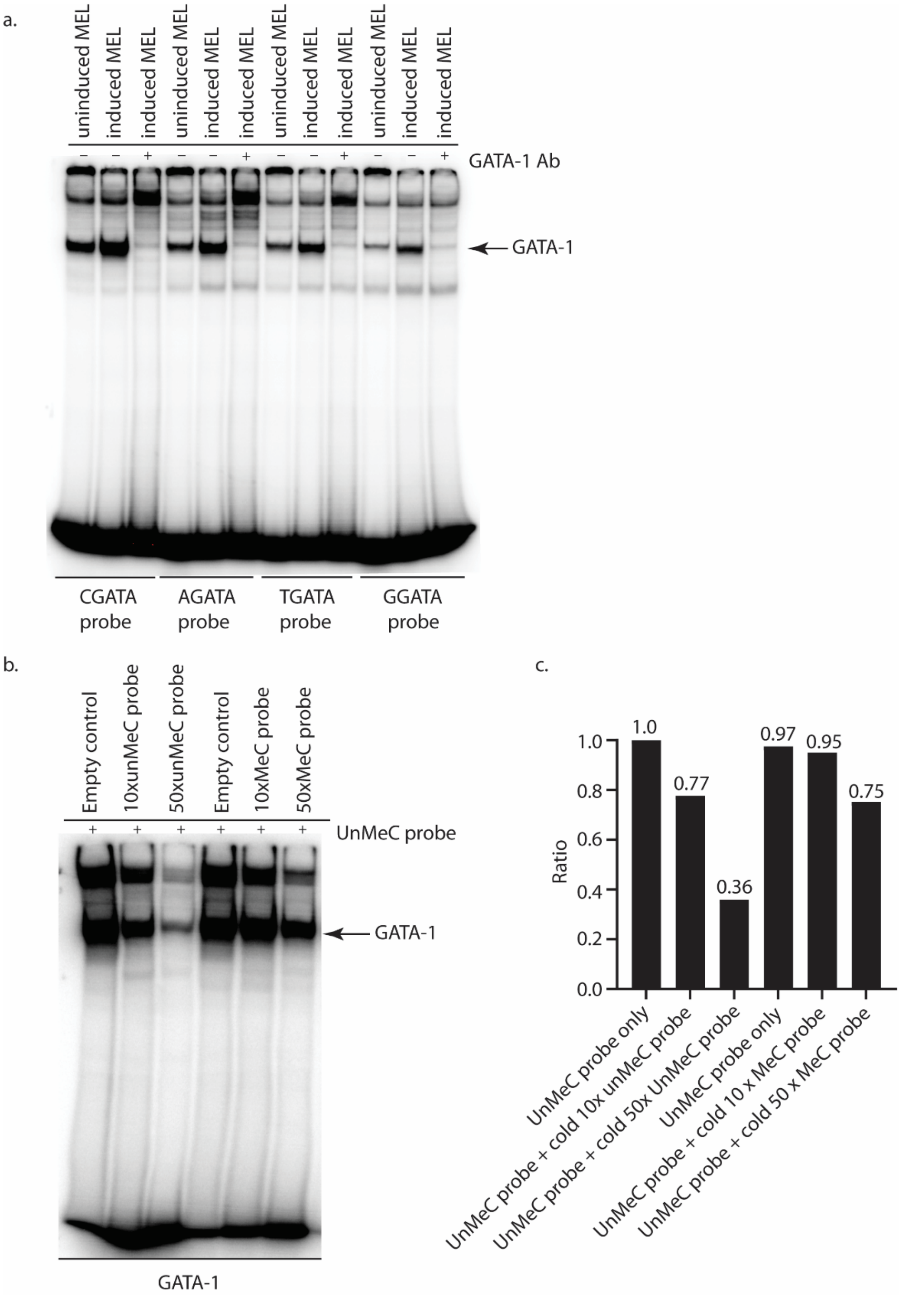

Supplementary Figure 1: **The preference binding of endogenous GATA-1 from erythroid cells to unmethylated CGATA probes *in vitro***

Electrophoretic mobility shift assays (EMSAs) assessing the ability of full-length mouse GATA-1 endogenously expressed in MEL cells (uninduced and induced with DMSO for 24 hours) to bind to probes containing CGATA, AGATA, TGATA and CGATA motifs (a), unmethylated and methylated CGATA probe with a cold unmethylated CGATA probe competition in induced MEL cells (b). EMSAs were repeated 2 times independently with similar results. Densitometry shows the competition between hot unmethylated/methylated CGATA probes with a cold unmethylated CGATA probe (c). Source data are provided as a Source Data file. (related to Figure 1)

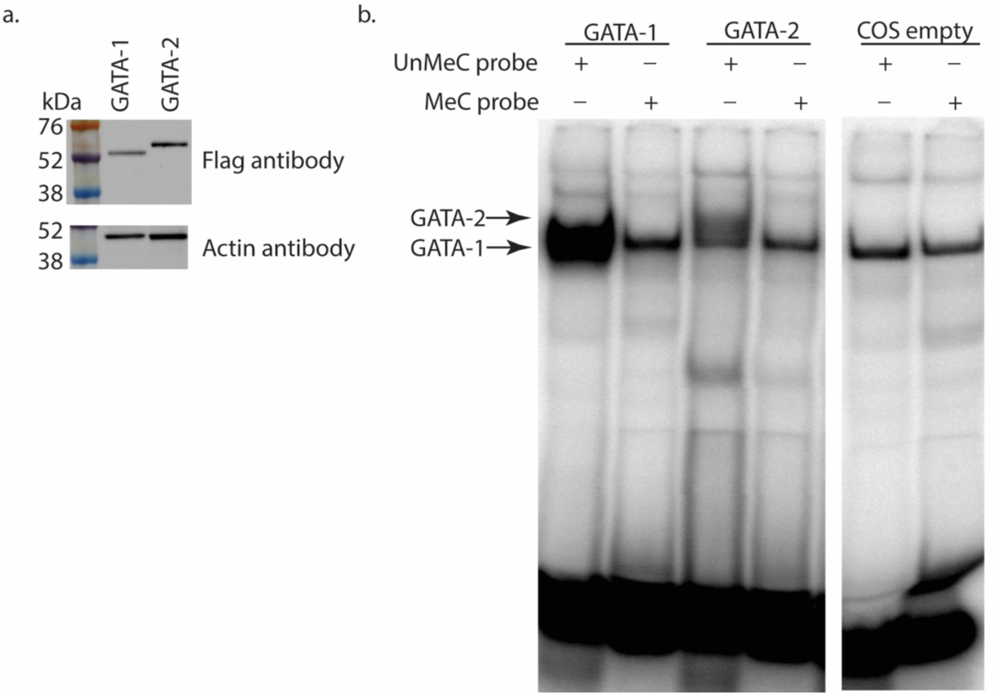

Supplementary Figure 2: **The binding affinity of GATA-2 and GATA-1 to unmethylated/ methylated CGATA probes**.

Electrophoretic mobility shift assays (EMSAs) assessing the ability of full-length mouse GATA-1 and GATA-2 overexpressed in COS cells with a flag tag (a), to bind to probes containing unmethylated/methylated CGATA (b). Western blot and EMSAs were repeated 2 times independently with similar results. Untransfected COS cells are shown as controls (b, right hand two lanes). Source data are provided as a Source Data file. (related to Figure 1)

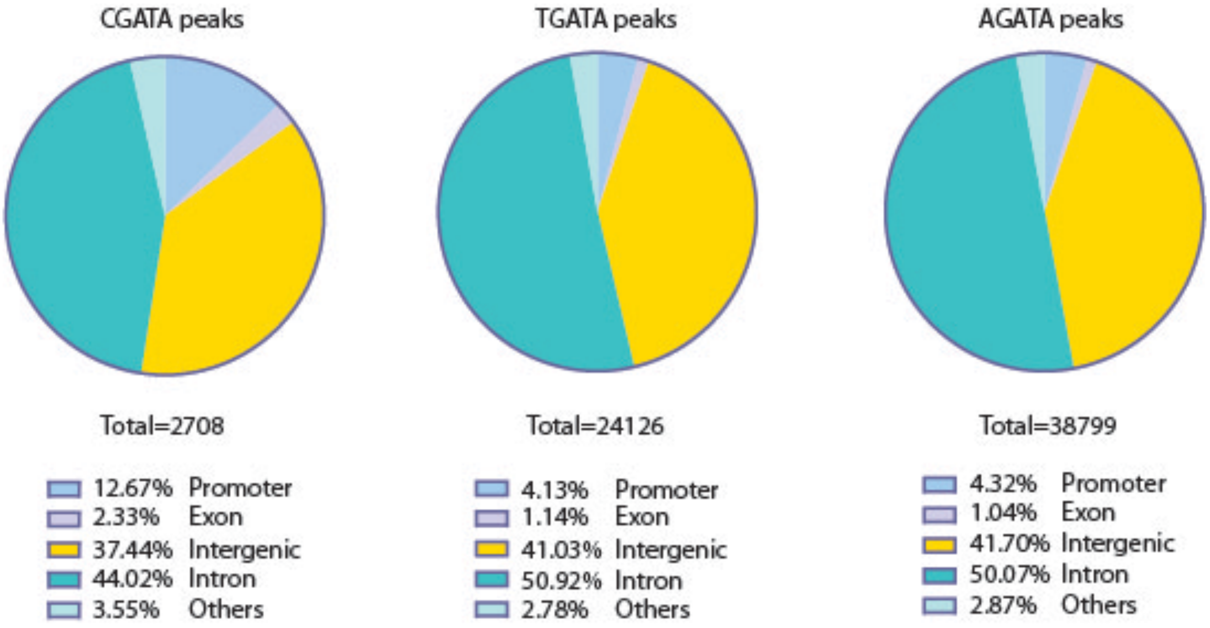

Supplementary Figure 3: **Genomic localization of GATA-1 peaks** [1].

The number of GATA-1 ChIP-seq peaks containing CGATA, TGATA and AGATA motifs near the center of the peak are 2708, 24126 and 38799 respectively. The distribution of CGATA, TGATA and AGATA motifs in GATA-1 ChIP-seq peaks throughout the genome are shown in each pie chart. (related to Figure 2)

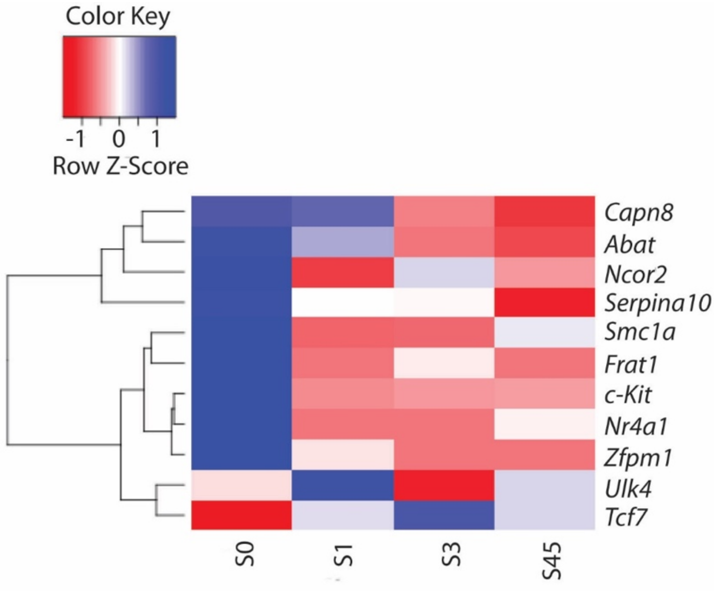

Supplementary Figure 4: **DNA methylation level of target genes during erythroblast differentiation of GATA-1 targets containing CGATA sites [2]**.

At selected CGATA sites, the methylation level decreased as differentiation progressed. Subsets S0, S1, S3 and S45 were increasingly differentiated erythroid progenitors sorted by flow cytometry from E12.5 to E13.5 Balb/C mouse embryos [4]. (related to Figure 2)

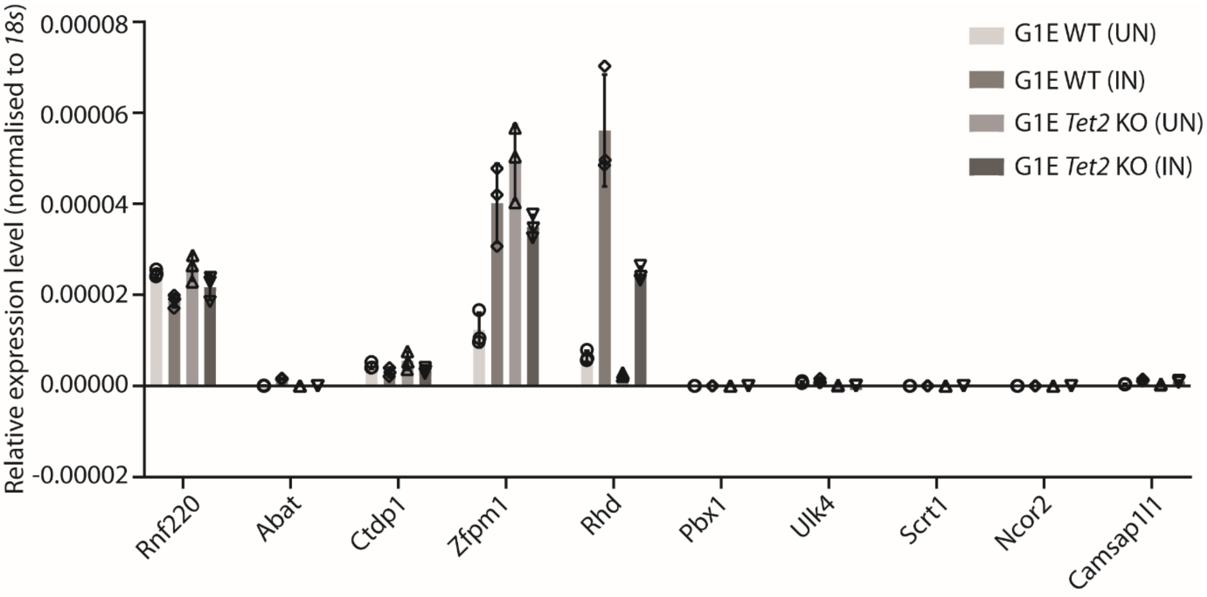

Supplementary Figure 5: **The gene expression level of GATA-1 target genes in G1E-ER4 cells.**

The expression level of target genes (those occupied by GATA-1 containing CGATA sites) in G1E WT and G1E *Tet2* KO4 cells by qPCR. Expressed relative to *18S* levels, (n=3 biologically independent cells, mean +/- standard deviation). Cells were induced with the addition of tamoxifen for 24 hours to restore GATA-1-ER fusion proteins in cell nucleus (IN). Cells incubated with ethanol were used as mock control (UN). Source data are provided as a Source Data file. (related to Figure 2)

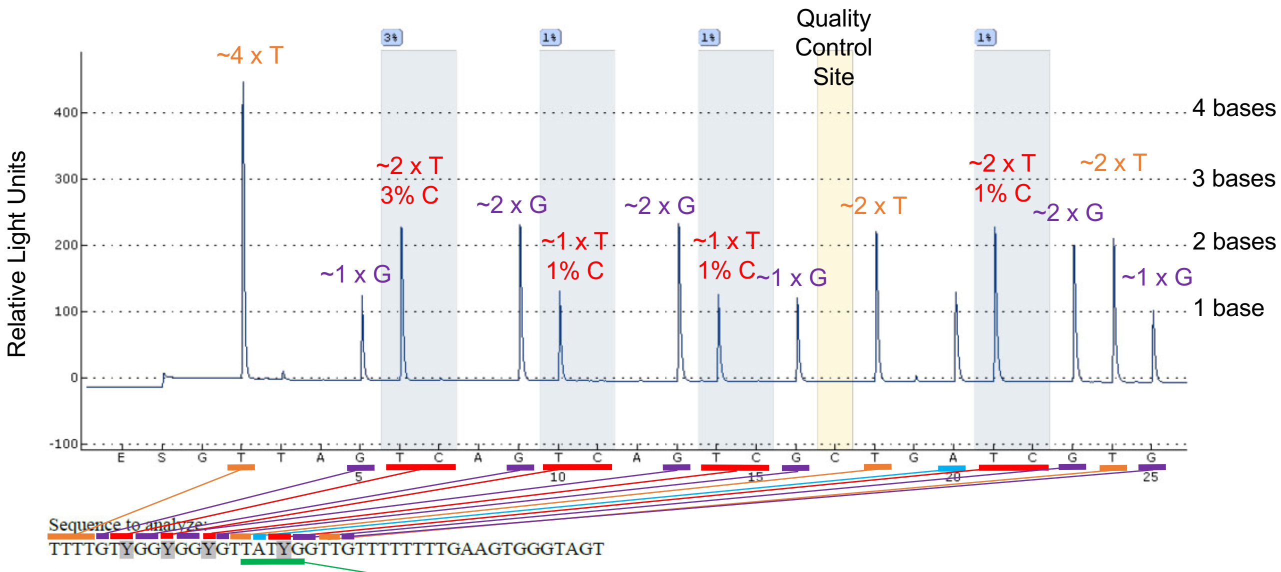

Position	1	2	3	4
Quality	Passed	Passed	Passed	Passed
Meth (%)	3	1	1	1

Supplementary Figure 6: **Hypomethylation of CGATA at intron 2 of *c-Kit* gene in MEL cells.**

Pyrosequencing to assess the DNA methylation level of the *c-Kit* gene at the intron 2 region in MEL cells. Annotated raw data is shown. Y = C or T. Blue boxes = G bases (reverse complement) in CG motifs susceptible to methylation. Bisulfite conversion leads to deamination of unmethylated cytosines to uracils (read as T), while methylated cytosines remain unchanged. Thus, the relative height of the C signal detected at the right-hand position of each blue box indicates the level of methylation that was present in the DNA at this site. The percentage shown at the top of each blue box shows percentage of methylation at this site, calculated using the relative height of the signal at the C position and the height of signal from a single T at the T position (0% = fully unmethylated, 100% = fully methylated). Yellow box = built-in quality control site consisting of cytosine (C) converted to thymine (T), demonstrating full bisulfite conversion of the treated DNA as no C signal could be detected here. Position 4 (the right hand blue shaded region) represents the CGATA site at intron 2 of *c-Kit* gene. Methylation across all four positions is very low, at 1-3%. (related to Figure 3).

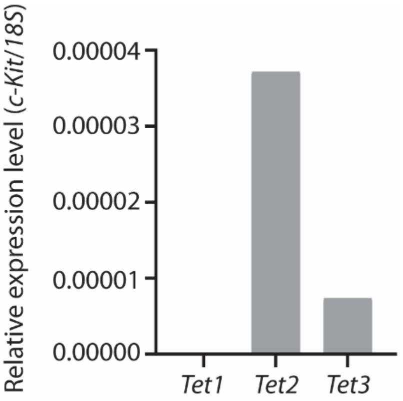

Supplementary Figure 7: ***Tet2* is highly expressed in G1E-ER4 cells.**
Expression of *Tet1*, *Tet2* and *Tet3* was examined by qPCR and is shown relative to *18S* levels. n = 1. Source data are provided as a Source Data file. (related to Figure 4)

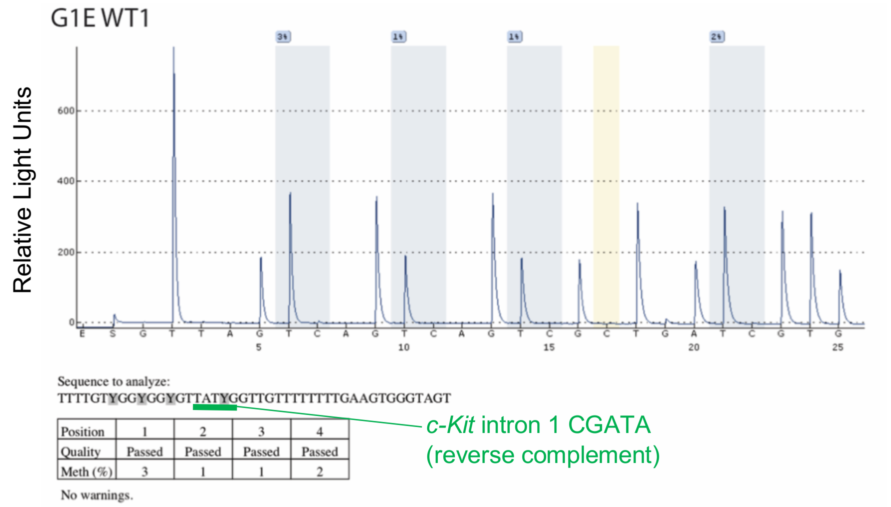

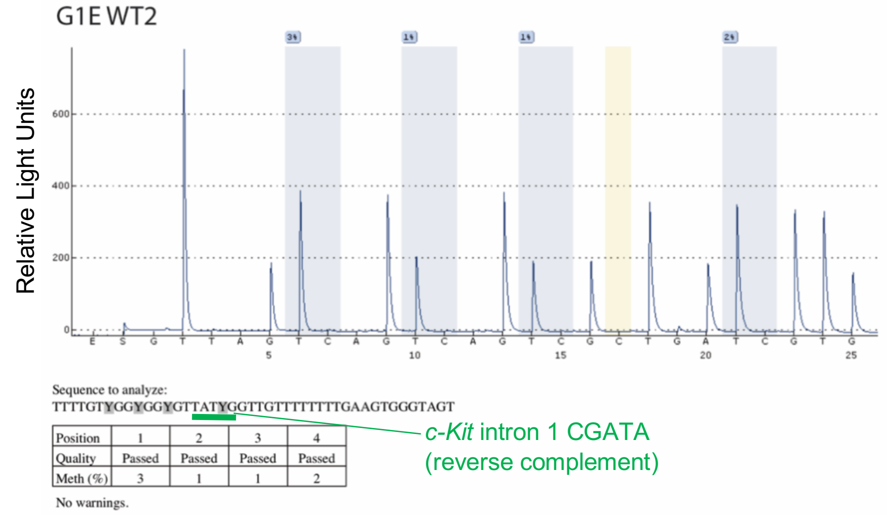

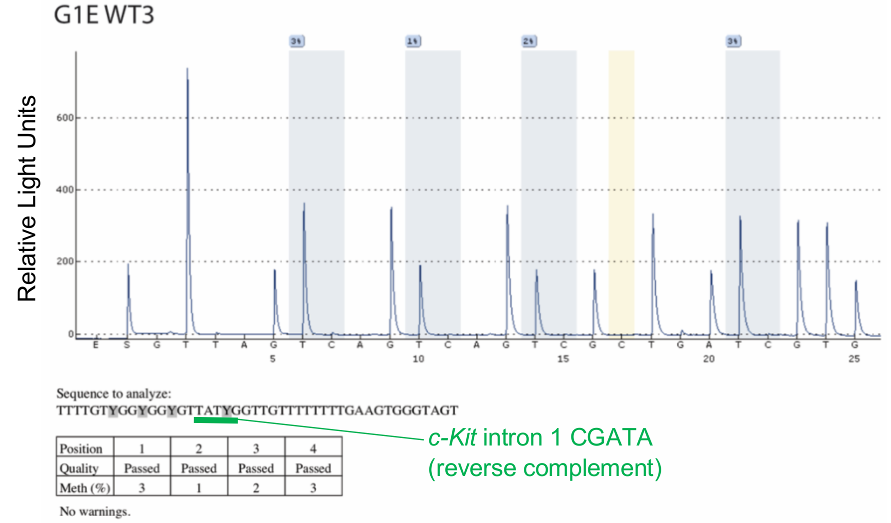

Supplementary Figure 8: Hypomethylation of CGATA at intron 2 of the *c-Kit* gene in wildtype G1E-ER4 cells.
Pyrosequencing to assess the DNA methylation level of the *c-Kit* intron 2 CGATA region in wildtype G1E-ER4 cells. Raw data is shown (see Supplementary Figure 6 for a full annotation). Y = C or T. Blue boxes = G bases (reverse complement) in CG motifs susceptible to methylation. Bisulfite conversion leads to deamination of unmethylated

cytosines to uracils (read as T), while methylated cytosines remain unchanged. Thus, the relative height of the C signal detected at the right-hand position of each blue box indicates the level of methylation that was present in the DNA at this site. The percentage shown at the top of each blue box shows percentage of methylation at this site, calculated using the relative height of the signal at the C position and the height of signal from a single T at the T position (0% = fully unmethylated, 100% = fully methylated). Yellow box = built-in quality control site consisting of cytosine (C) converted to thymine (T), demonstrating full bisulfite conversion of the treated DNA as no C signal could be detected here. Position 4 (the right hand blue shaded region) represents the CGATA site at intron 2 of *c-Kit* gene. Methylation across these four positions is very low, at 1-3%. (related to Figure 4)

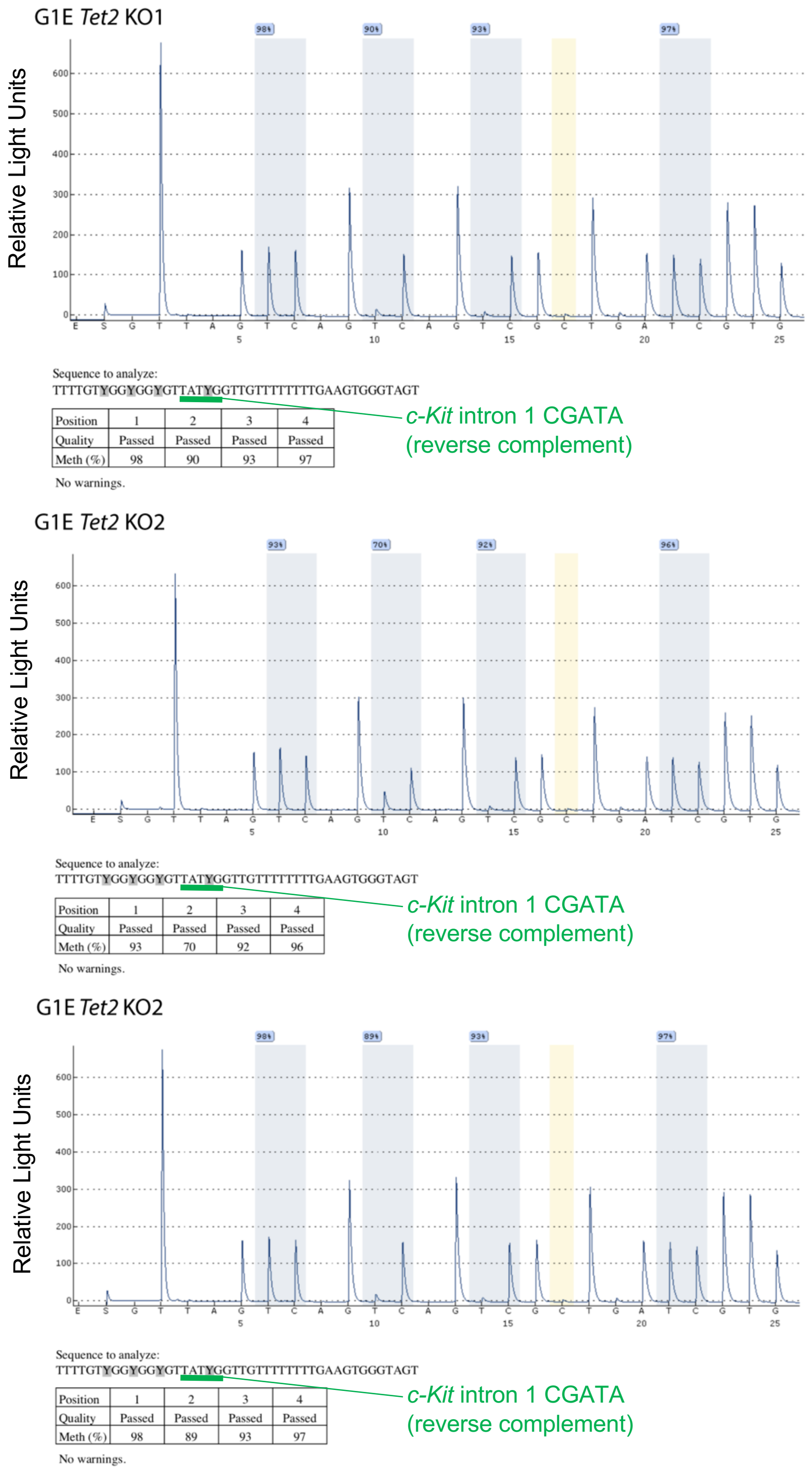

Supplementary Figure 9: Hypermethylation of CGATA at intron 2 of the *c-Kit* gene in G1E-ER4 *Tet2* knockout cells.

Pyrosequencing to assess the DNA methylation level of the *c-Kit* intron 2 CGATA region in G1E-ER4 *Tet2* knockout cells. Raw data is shown (see Supplementary Figure 6 for a full annotation). Y = C or T. Blue boxes = G bases (reverse complement) in CG motifs susceptible to methylation. Bisulfite conversion leads to deamination of unmethylated cytosines to uracils (read as T), while methylated cytosines remain unchanged. Thus, the

relative height of the C signal detected at the right-hand position of each blue box indicates the level of methylation that was present in the DNA at this site. The percentage shown at the top of each blue box shows percentage of methylation at this site, calculated using the relative height of the signal at the C position and the height of signal from a single T at the T position (0% = fully unmethylated, 100% = fully methylated). Yellow box = built-in quality control site consisting of cytosine (C) converted to thymine (T), demonstrating full bisulfite conversion of the treated DNA as no C signal could be detected here. Position 4 (the right hand blue shaded region) represents the CGATA site at intron 2 of *c-Kit* gene. Methylation across these four positions is very high, at 70-98%. (related to Figure 4)

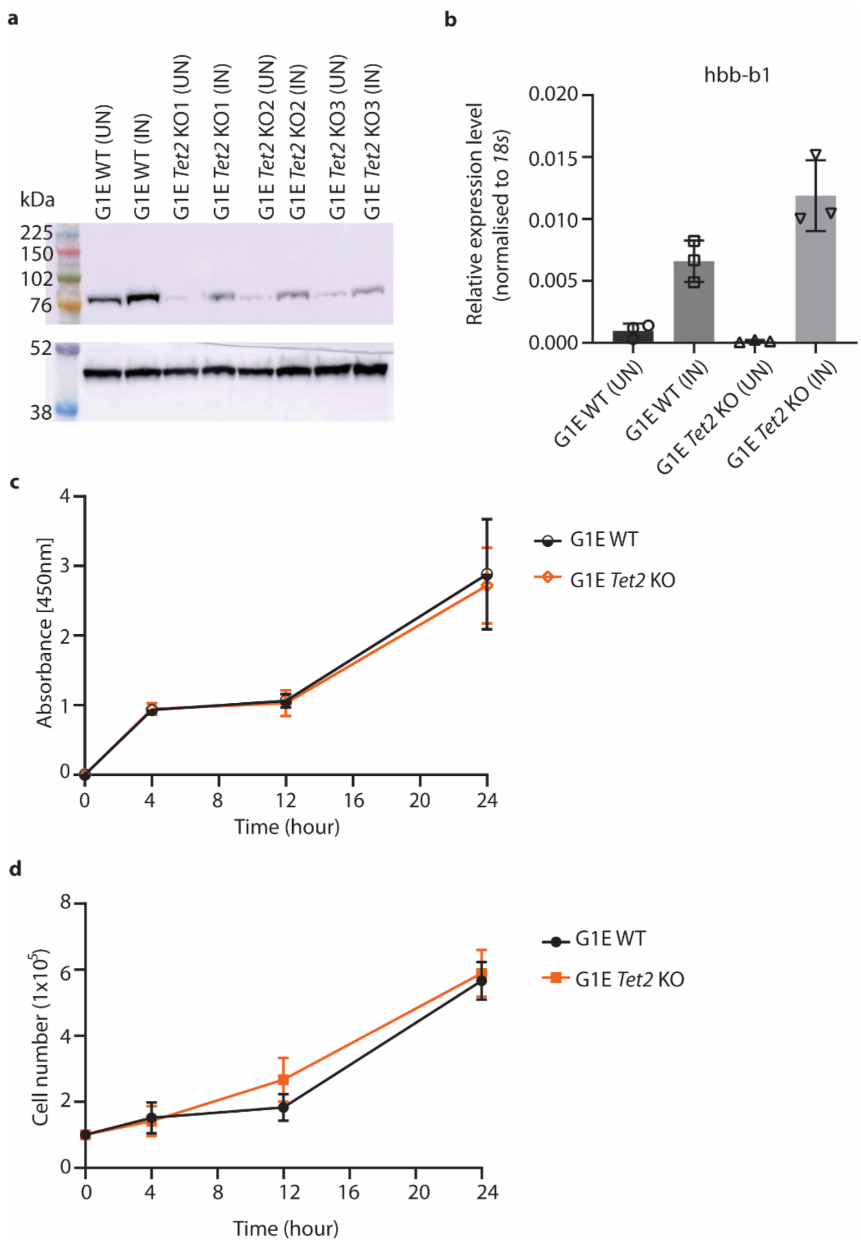

Supplementary Figure 10: G1E-ER4 cells with *Tet2* knock out are still able to be induced with tamoxifen and have no detectable differences in differentiation or proliferation. (a). Western blot using an antibody to GATA-1 shows the nuclear protein level of GATA-1 in WT and *Tet2* KO G1E-ER4 cell nucleus after incubating with ethanol vehicle (UN) and tamoxifen (IN) for 24 hours. A blot probed with an antibody to actin is shown as a loading control.The experiment was repeated independently 2 times with similar results. (b). *hbb-b1* expression level, showing successful induction of haemoglobin following GATA-1 nuclear localisation, in WT and *Tet2* KO G1E-ER4 cells after incubating with vehicle (UN) and tamoxifen (IN) for 24 hours. Cell proliferation of WT and *Tet2* KO G1E-ER4 cells as determined by a BrdU cell proliferation assay (c) and cell number counting (d) over a 24 hour time course suggests that *Tet2* KO has no measurable impact on cell proliferation or survival. (n=3 biologically independent cells, mean +/- standard deviation). Source data are provided as a Source Data file. (related to Figure 4)

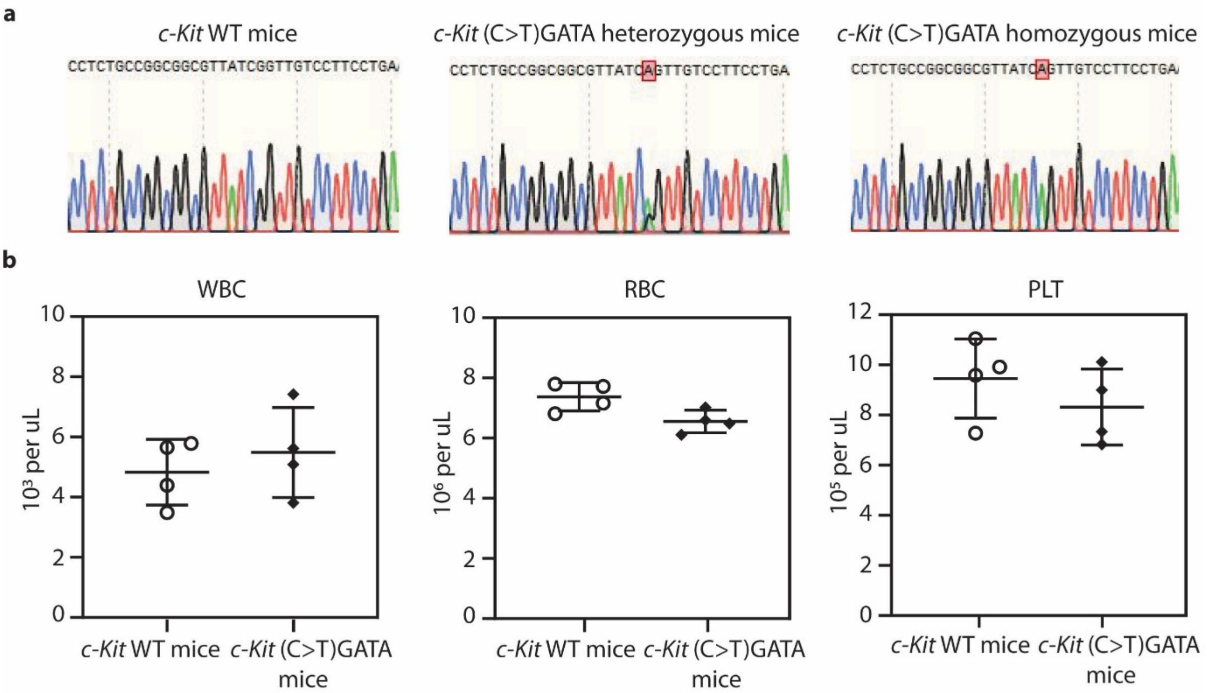

Supplementary Figure 11: Genotyping and peripheral blood counts in *c-Kit* wild type and (C>T)GATA mutant mice.

(a) Illustrative mouse genotyping by Sanger sequencing. Wild type and (C>T)GATA homozygous littermate mutant mice were used for experiments. Heterozygous mice were used for breeding. (b) White blood cell (WBC), Red blood cell (RBC) and Platelet (PLT) blood counts in peripheral blood from wild type and (C>T)GATA homozygous littermate mutant mice. Blood was collected and analysed using a Sysmex XN-1000RF. n=4 biologically independent animals, mean +/- standard deviation. Measurements were taken from distinct samples (animals). Source data are provided as a Source Data file. (related to Figure 5)

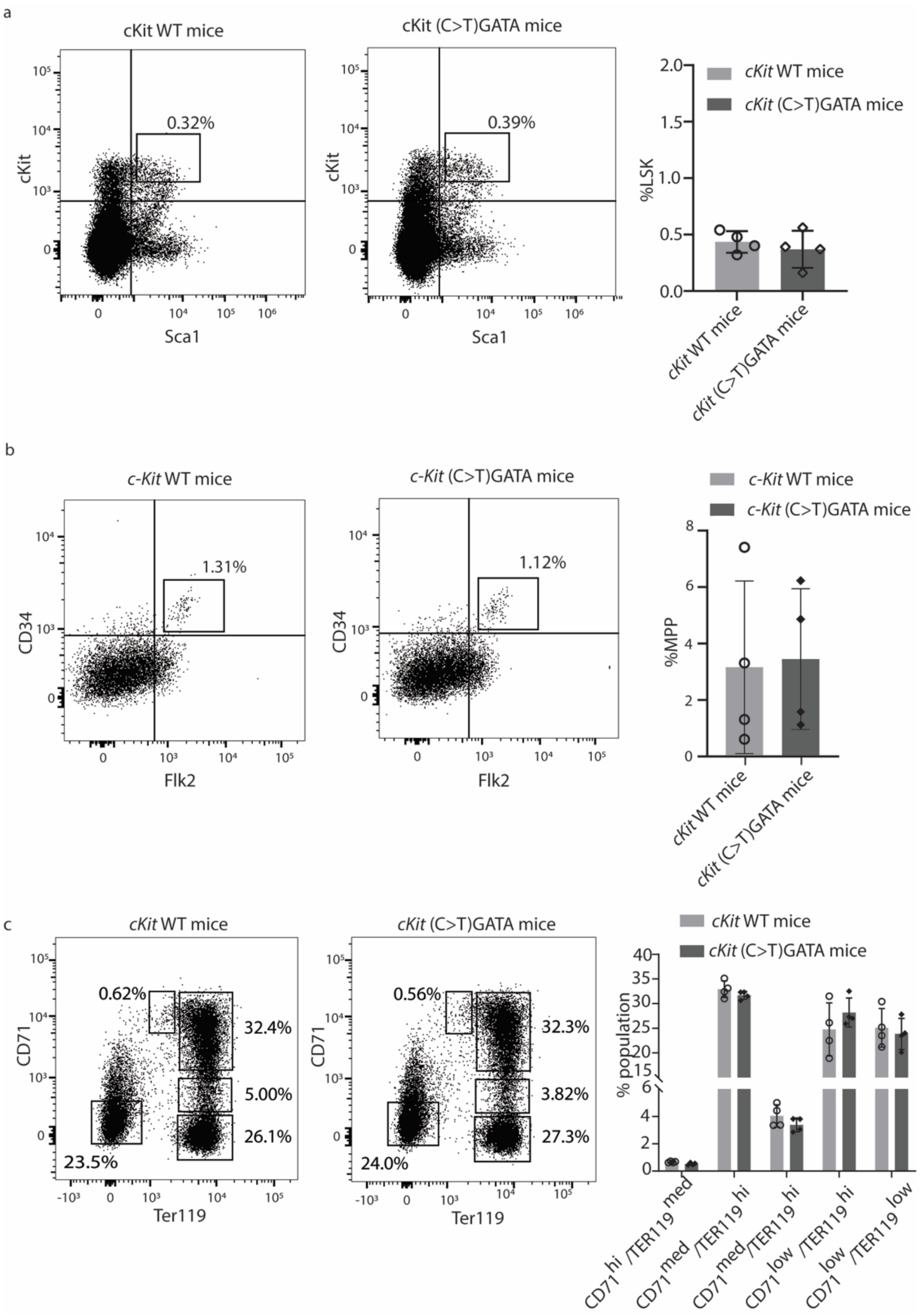

Supplementary Figure 12: **The impact of the *c-Kit*(C>T)GATA mutation on hematopoiesis and erythropoiesis.**

Bone marrow cells from WT and homozygous *c-Kit*(C>T)GATA mutant mice were stained with cKit and Sca1 (a), CD34 and Flk2 (b), CD71 and Ter119 (c). Lineage negative, cKit positive and Sca1 positive cells are hematopoietic stem cells (LSKs). Lineage negative, CD34 positive and Flk2 positive cells are multipotent progenitors (MPPs). $CD71^{low}Ter119^{low}$ cells are non-erythroid cells, $CD71^{hi}Ter119^{med}$ cells are proerythroblasts, $CD71^{hi}Ter119^{hi}$ cells are basophilic erythroblasts, $CD71^{med}Ter119^{hi}$ cells are polychromatophilic erythroblasts, $CD71^{low}Ter119^{hi}$ cells are more mature orthochromatophilic erythroblasts and reticulocytes. (n=4 biologically independent animals, mean +/- standard deviation). Source data are provided as a Source Data file. (related to Figure 5)

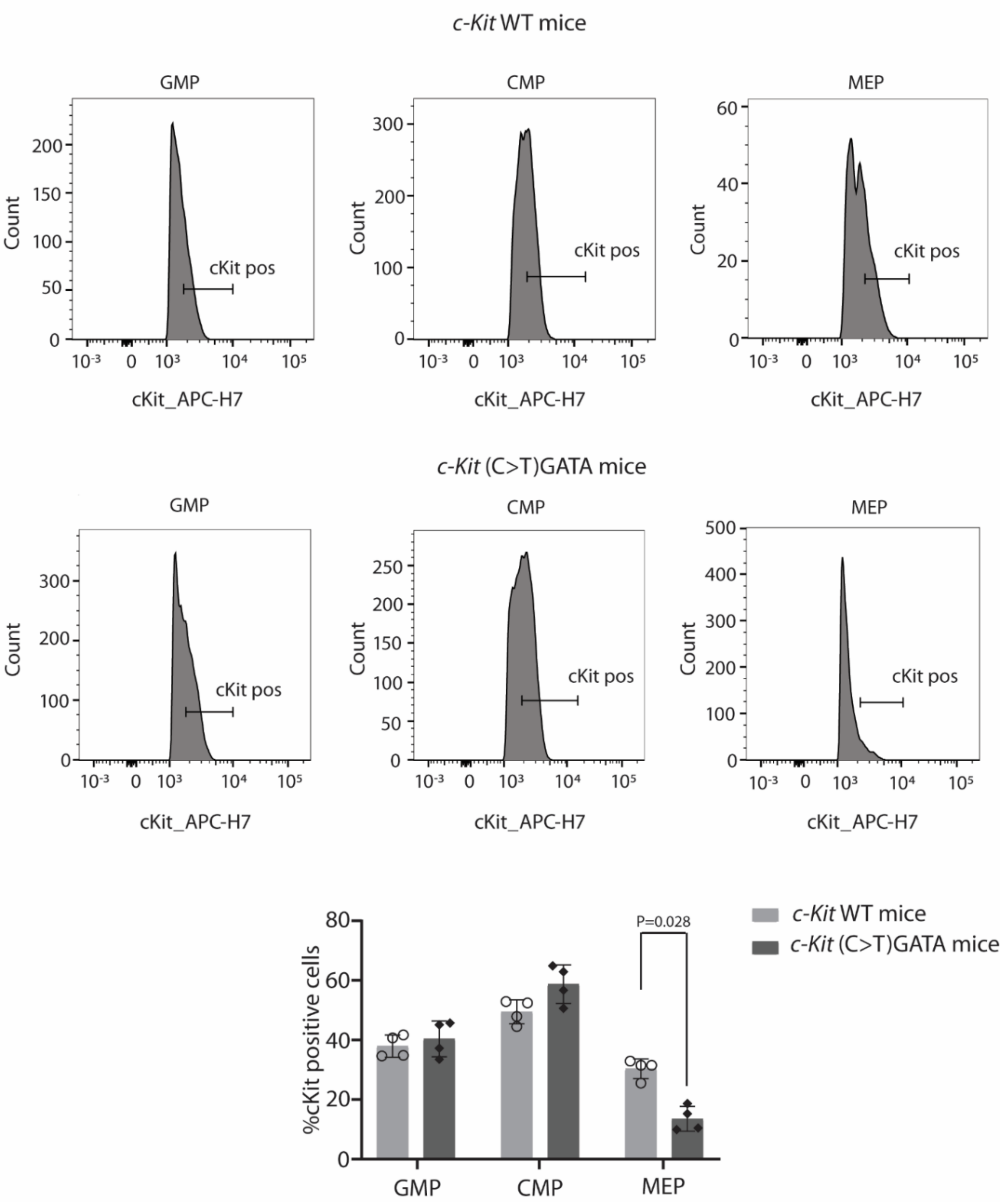

Supplementary Figure 13: **Increased proportions of low c-Kit expressing megakaryocyte-erythroid progenitors (MEPs) in *c-Kit* (C>T)GATA mice.**

The expression level of cKit in granulocyte-monocyte progenitors (GMPs), common myeloid progenitors (CMPs) and megakaryocyte-erythrocyte progenitors (MEPs) were assessed in wild type and *c-Kit* (C>T)GATA mutant mice by flow cytometry. The percentage of cKit positive GMPs, CMPs and MEPs in wild type and mutant mice were summarised in the histogram (n=4 biologically independent animals, mean +/- standard deviation). A Mann-Whitney U non-parametric, 2-tailed statistical test was used where a P value of less than 0.05 indicates a significant difference between the two means. Adjustments were not made for multiple comparisons. Source data are provided as a Source Data file. (related to Figure 5)

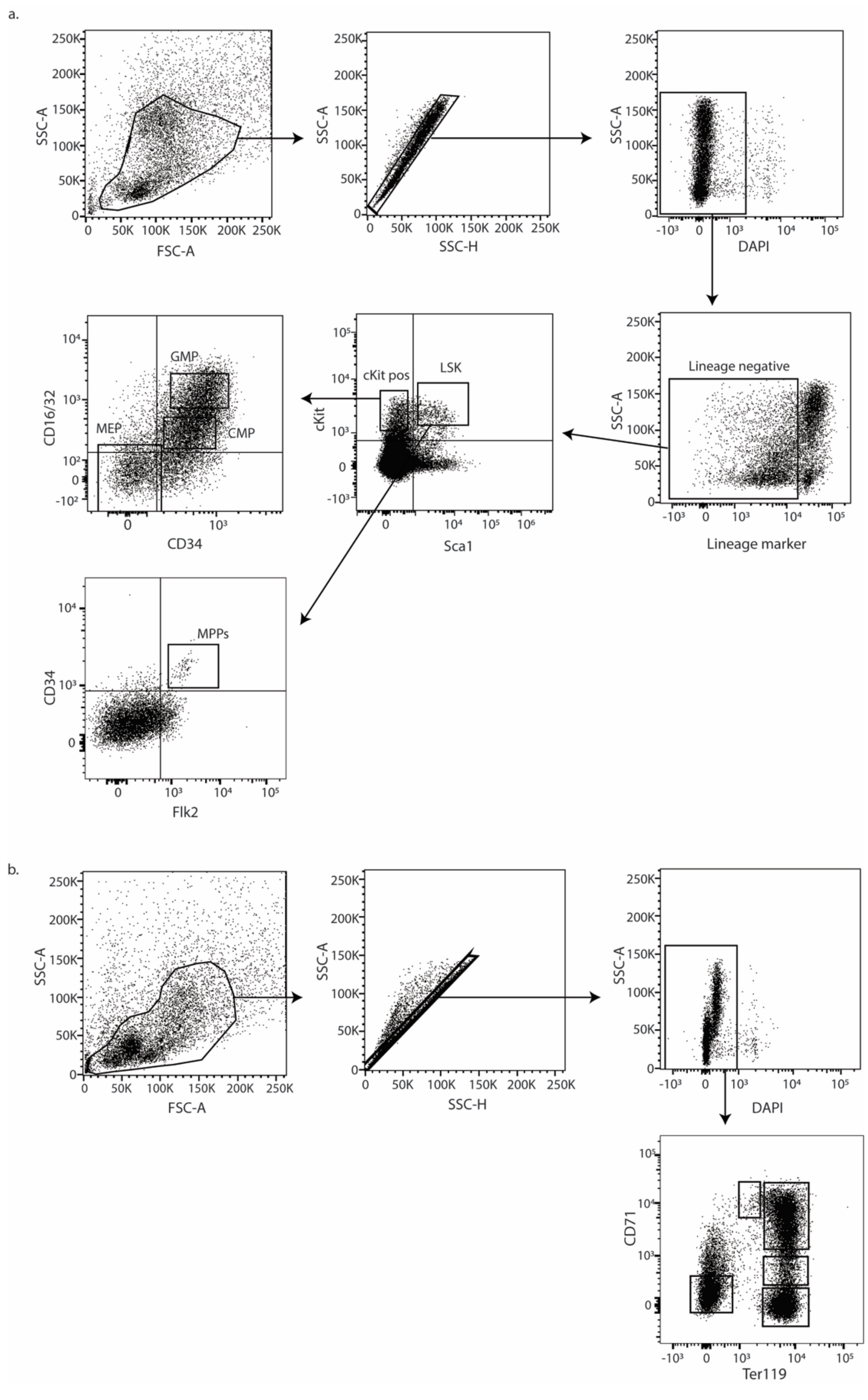

Supplementary Figure 14: Flow cytometry gating strategies.

Flow cytometry gating strategies for haematopoietic stem cell and progenitor cells (HSPCs) (a: Fig. 2e, Fig. 5, Supplementary Figure 12 and Supplementary Figure 13) and erythrocyte populations (b: Fig. 2e and Supplementary Figure 12). All antibodies have been listed in the methods section.

Supplementary Table 1: The genomic localisation of CGATA sites in target genes

Gene Name	Chromosome	Start	end
Camsap1l1	chr1	138242575	138242580
Pbx1	chr1	170218574	170218579
Scrt1	chr15	76354197	76354202
Abat	chr16	8530213	8530218
Ctdp1	chr18	80580992	80580997
Rnf220	chr4	116985190	116985195
Rhd	chr4	134425669	134425674
Ncor2	chr5	125620949	125620954
c-Kit	chr5	76004254	76004259
Zfpm1	chr8	124820443	124820448
Ulk4	chr9	121194952	121194957

Supplementary Table 2: Raw data for methylation level of *c-Kit* intron 2 CGATA site during hematopoiesis (published publicly available data [22], data source: http://www.medical-epigenomics.org/papers/broad_mirror/invivomethylation/index.html)

Sample identifier	Chromosome	Start	End	Annotation	Percentage of methylation
HSC replicate 1	chr5	76004258	76004259	Kit	54.5
HSC replicate 2	chr5	76004258	76004259	Kit	92.3
MPP replicate 1	chr5	76004258	76004259	Kit	66.6
Mpp replicate 2	chr5	76004258	76004259	Kit	86.2
CMP replicate 1	chr5	76004258	76004259	Kit	78.4
CMP replicate 2	chr5	76004258	76004259	Kit	83.3
MEP replicate 1	chr5	76004258	76004259	Kit	3
MEP replicate 2	chr5	76004258	76004259	Kit	10
Eryth replicate 1	chr5	76004258	76004259	Kit	0
Eryth replicate 2	chr5	76004258	76004259	Kit	0

Supplementary Table 3: EMSA probes

Probe	Fwd(F)/ Rev(R)	5'-Sequence-3'
CGATA probe (UnMeC probe)	F	ATGATG**CGATA**ATTATGATG
	R	CATCATAAT**TATCG**CATCAT
AGATA probe	F	ATGATG**AGATA**ATTATGATG
	R	CATCATAAT**TATCT**CATCAT
TGATA probe	F	ATGATG**TGATA**ATTATGATG
	R	CATCATAAT**TATCA**CATCAT
GGATA probe	F	ATGATG**GGATA**ATTATGATG
	R	CATCATAAT**TATCC**CATCAT
Methylated CGATA probe (MeC probe)	F	ATGATG**[5MeC]GATA**ATTATGATG
	R	CATCATAAT**TAT[5MeC]G**CATCAT
Hemi-methylated probe 1 (HemiMeC probe1)	F	ATGATG**[5MeC]GATA**ATTATGATG
	R	CATCATAAT**TATCG**CATCAT
Hemi-methylated probe 2 (HemiMeC probe2)	F	ATGATG**CGATA**ATTATGATG
	R	CATCATAAT**TAT[5MeC]GC**ATCAT

Supplementary Table 4: qPCR primers

Gene	Fwd (F)/ Rev (R)	5'-Sequence-3'
18S	F	CACGGCCGGTACAGTGAAAC
	R	AGAGGAGCGAGCGACCAA
c-Kit	F	TCGCACGGGCACATACAC
	R	AAAGTTTGGCAGGATCTCTAACAAA
Rnf220	F	ATGTGCAGTGGCAAAGAGA
	R	ACATCAGCCTCCGTGTATTG
*Aba*t	F	GGGCCTAGATCTAAGGAACTAATG
	R	CTAGGTAGTTACCTCTGCTCTCT
Ctdp1	F	TTCTGGTGAGATTGGAAGGATG
	R	AAACCGTCGCTGTGGAAA
Zfpm1	F	CAAGTCCACCCAGAGAAGATG
	R	GCCTCATCTCCAACTCCTG
Rhd	F	GAACAATCTTGCTGGACCATTTC
	R	GCTGAGATCAGCACAGGTAAG
Pbx1	F	GAATGAAGCCTGCCTTGTTTAAT
	R	TAGCAGCATGTTGTCCAGTC
Ulk4	F	GTACGAGACCAGCAATCATCTC
	R	CCAAACTCTCTCACGACATCTT
Scrt1	F	GGAGTGCGACTGCAAGATAA
	R	ACAGCCGCAGCATACATAG
Ncor2	F	GATGCGGAAGAAGCTGATCT
	R	CCTTCGCGGATTGTTCTCTAT
Camsap1l1	F	CCAGGAGAAACTGGTAACTGAA
	R	TCTCTACAGTGTAAGCCATCATTAG

Supplementary Table 5: ChIP-qPCR primers

Gene	Fwd(F)/Rev(R)	5'-Sequence-3'
c-Kit +5kb	F	TAAGGGAGACTGCGTGAGAT
	R	GGGCCACATTTCTACCTTCTT
c-Kit +7kb	F	GTGAGTGGAGTGCCAAGATAA
	R	GATTCCACCACAAGGGAGAC
c-Kit +33kb	F	AAGATGGCCGGGAAATTGA
	R	CTGAGCATGTCTCCTCAGC
c-Kit +49kb	F	GACTCTAAGCGGGTTGTAGATG
	R	CAGGGACTCATGGGAGAGATA
c-Kit +58kb	F	GTGGGAGGAGTTAGGGAATATG
	R	GCATTTCACCGCAAGAGATAC
Klf1	F	AGCACACCACACACATATCG
	R	ATGGGCTATGAGGCTAGGAA
Negative control	F	GGTCTGGAGAAGTAGCTCAGG
	R	GAGGTGGGAGCTGGAATCTG

Supplementary Table 6: Pyrosequencing primers

Gene	Id	5'-Sequence-3'
c-Kit	Fwd	GAGGGGTTAATAAAGTTGTTTTTGAAGAT
	Rev	ATATCTCCTCAACTACCCACTTC
	Probe	GGGAAATTGATTGTG

Supplementary Table 7: Single guide RNAs (sgRNAs) for genome editing in mouse cell lines

Gene	Fwd(F)/Rev(R)	5'-Sequence-3'
Tet2 coding region sgRNA	F	CACCGTGCCATTTGGCATGTTCTGC
	R	AAACGCAGAACATGCCAAATGGCAC
c-Kit Intron 2 CGATA region sgRNA	F	CACCGGCCTCTGCCGGCGGCGTTAT
	R	AAACATAACGCCGCCGGCAGAGGCC
c-Kit donor	F	CTCAAAGAGGGGTTAATAAAGCTGCC TCTGAAGATGGCCGGGAAATTGACT GTGCCTCTGCCGGCGGCG**TTATAA** GTTGTCCTTCCTGAAGTGGGCAGCT GAGGAGACATGCTCAGTGATAAGCCC CAGCTTAGCTTCAGCTTCAGAAATGTT GGG

Supplementary Table 8: Single guide RNA (sgRNA) *In vitro* transcription primers for CRISPR mouse generation

Name	Fwd(F)/ Rev(R)	5'-Sequence-3'
sgRNA#1	F	TTAATACGACTCACTATAGAGGACAACCGATAACGCC GCGTTTTAGAGCTAGAAATAGCAAGTTAAAATAAGGC TAGTC
	R	AAAAGCACCGACTCGGTGCC
sgRNA#2	F	TTAATACGACTCACTATAGATCGGTTGTCCTTCCTGAA G GTTTTAGAGCTAGAAATAGCAAGTTAAAATAAGGCTA GTC
	R	AAAAGCACCGACTCGGTGCC
c-Kit donor	F	CTCAAAGAGGGGTTAATAAAGCTGCCTCTGAAGATGG CCGGGAAATTGACTGTGCCTCTGCCGGCGGCGT**TATCA** GTTGTCCTTCCTGAAGTGGGCAGCTGAGGAGACATGCT CAGTGATAAGCCCCAGCTTAGCTTCAGAAATGTTGGG
Genotyping primers	F	GGGTTGGTTCTTTTCAGCAA
	R	CAATCACCAGGGCATAGGAG

Supplementary Table 9: Public Dataset Accessions

Cell Type	Method	Accession [hyperlink]
MEL	ChIP-Seq (GATA-1)	ENCSR000ETA [https://www.encodeproject.org/experiments/ENCSR000ETA/]
MEL	ChIP-Seq (GATA-1)	ENCSR000EUG [https://www.encodeproject.org/experiments/ENCSR000EUG/]
Erythroblast (E14.5)	ChIP-Seq (GATA-1)	ENCSR000DIL [https://www.encodeproject.org/experiments/ENCSR000DIL/]
G1E	ChIP-Seq (GATA-1)	ENCSR000DIC [https://www.encodeproject.org/experiments/ENCSR000DIC/]
G1E-ER4 (0h)	ChIP-Seq (GATA-1)	ENCSR000DHC [https://www.encodeproject.org/experiments/ENCSR000DHC/]
G1E-ER4 (3h)	ChIP-Seq (GATA-1)	ENCSR000DGY [https://www.encodeproject.org/experiments/ENCSR000DGY/]
G1E-ER4 (7h)	ChIP-Seq (GATA-1)	ENCSR000DGX [https://www.encodeproject.org/experiments/ENCSR000DGX/]
G1E-ER4 (14h)	ChIP-Seq (GATA-1)	ENCSR000DIQ [https://www.encodeproject.org/experiments/ENCSR000DIQ/]

G1E-ER4 (24h)	ChIP-Seq (GATA-1)	ENCSR000DHA [https://www.encodeproject.org/experiments/ENCSR000DHA/]
G1E-ER4 (30h)	ChIP-Seq (GATA-1)	ENCSR000DGZ [https://www.encodeproject.org/experiments/ENCSR000DGZ/]
Blood cells	RRBS	GSE38557 [https://www.ncbi.nlm.nih.gov/geo/query/acc.cgi?acc=GSE38557]
Sorted foetal liver cells	RRBS	GSE32214 [https://www.ncbi.nlm.nih.gov/geo/query/acc.cgi?acc=GSE32214]
Megakaryocyte embryo 14.5	ChIP-Seq (H3K4me1)	ENCFF213NED [https://www.encodeproject.org/experiments/ENCSR000DHR/]
MEL	ChIP-Seq (H3K4me3)	ENCFF335CVP [https://www.encodeproject.org/experiments/ENCSR000CEX/]
MEL	ChIP-Seq (H3K27ac)	ENCFF352TNV [https://www.encodeproject.org/experiments/ENCSR000CEV/]
MEL	DNase-Seq	ENCFF155MQS [https://www.encodeproject.org/experiments/ENCSR446MUM/]
Megakaryocyte-erythroid progenitor cell	ATAC-Seq	ENCFF831CZO [https://www.encodeproject.org/experiments/ENCSR064IHX/]

Supplementary References:

1. Yue, F. *et al.* A comparative encyclopedia of DNA elements in the mouse genome. *Nature* **515**, 355-364 (2014).
2. Shearstone, J.R. *et al.* Global DNA demethylation during mouse erythropoiesis in vivo. *Science* **334**, 799-802 (2011).
