# Supplementary material for: Methylation of a CGATA element inhibits binding and regulation by GATA-1
Source: Nat Commun. 2020 May 22;11:2560. doi: 10.1038/s41467-020-16388-1 (PMC7244756; doi:10.1038/s41467-020-16388-1)
Supplement: Supplementary file 2 — Reporting summary [file 41467_2020_16388_MOESM2_ESM.pdf]

nature research

Corresponding author(s): Merlin Crossley

Last updated by author(s): Feb 21, 2020

Reporting Summary

Nature Research wishes to improve the reproducibility of the work that we publish. This form provides structure for consistency and transparency in reporting. For further information on Nature Research policies, see Authors & Referees and the Editorial Policy Checklist .

Statistics

For all statistical analyses, confirm that the following items are present in the figure legend, table legend, main text, or Methods section.

n/a	Confirmed	
☐	☒	The exact sample size (*n*) for each experimental group/condition, given as a discrete number and unit of measurement
☐	☒	A statement on whether measurements were taken from distinct samples or whether the same sample was measured repeatedly
☐	☒	The statistical test(s) used AND whether they are one- or two-sided<br>*Only common tests should be described solely by name; describe more complex techniques in the Methods section.*
☒	☐	A description of all covariates tested
☒	☐	A description of any assumptions or corrections, such as tests of normality and adjustment for multiple comparisons
☐	☒	A full description of the statistical parameters including central tendency (e.g. means) or other basic estimates (e.g. regression coefficient) AND variation (e.g. standard deviation) or associated estimates of uncertainty (e.g. confidence intervals)
☐	☒	For null hypothesis testing, the test statistic (e.g. *F*, *t*, *r*) with confidence intervals, effect sizes, degrees of freedom and *P* value noted<br>*Give P values as exact values whenever suitable.*
☒	☐	For Bayesian analysis, information on the choice of priors and Markov chain Monte Carlo settings
☒	☐	For hierarchical and complex designs, identification of the appropriate level for tests and full reporting of outcomes
☒	☐	Estimates of effect sizes (e.g. Cohen's *d*, Pearson's *r*), indicating how they were calculated

Our web collection on statistics for biologists contains articles on many of the points above.

Software and code

Policy information about availability of computer code

Data collection	All software used in this study for data collection are either commercially available or open source.
Data analysis	For ChIP-seq analysis:<br>Peak Analyzer (v1.4) was used to split broad peaks into narrow peaks and for peak annotation.<br>MEME ChIP (v4.9.1) was used for motif analysis.<br>FIMO (v5.1.1) was used for motif screening<br>For RRBS analysis:<br>RnBeads (v2.0) was used to analyse all single CpG methylation data<br>BEDtools (v2.26.0) was used to find DNA methylation level of "CGATA" peaks<br>gplot library (v1.11) was used to draw heatmaps of DNA methylation changes of "CGATA" peaks during differentiation<br>For Flow cytometry anaylsis:<br>BD FACS DiVa (v9.0)<br>FlowJo (v10.0)

For manuscripts utilizing custom algorithms or software that are central to the research but not yet described in published literature, software must be made available to editors/reviewers. We strongly encourage code deposition in a community repository (e.g. GitHub). See the Nature Research guidelines for submitting code & software for further information.

Data

Policy information about availability of data

All manuscripts must include a data availability statement. This statement should provide the following information, where applicable:

- Accession codes, unique identifiers, or web links for publicly available datasets
- A list of figures that have associated raw data
- A description of any restrictions on data availability

Accession codes, unique identifiers, or web links for publicly available datasets:
MEL ChIP-Seq (GATA-1) ENCSR000ETA [https://www.encodeproject.org/experiments/ENCSR000ETA/]
MEL ChIP-Seq (GATA-1) ENCSR000EUG [https://www.encodeproject.org/experiments/ENCSR000EUG/]
Erythroblast (E14.5) ChIP-Seq (GATA-1) ENCSR000DIL [https://www.encodeproject.org/experiments/ENCSR000DIL/]
G1E ChIP-Seq (GATA-1) ENCSR000DIC [https://www.encodeproject.org/experiments/ENCSR000DIC/]
G1E-ER4 (0h) ChIP-Seq (GATA-1) ENCSR000DHC [https://www.encodeproject.org/experiments/ENCSR000DHC/]
G1E-ER4 (3h) ChIP-Seq (GATA-1) ENCSR000DGY [https://www.encodeproject.org/experiments/ENCSR000DGY/]
G1E-ER4 (7h) ChIP-Seq (GATA-1) ENCSR000DGX [https://www.encodeproject.org/experiments/ENCSR000DGX/]
G1E-ER4 (14h) ChIP-Seq (GATA-1) ENCSR000DIQ [https://www.encodeproject.org/experiments/ENCSR000DIQ/]
G1E-ER4 (24h) ChIP-Seq (GATA-1) ENCSR000DHA [https://www.encodeproject.org/experiments/ENCSR000DHA/]
G1E-ER4 (30h) ChIP-Seq (GATA-1) ENCSR000DGZ [https://www.encodeproject.org/experiments/ENCSR000DGZ/]
Blood cells RRBS GSE38557 [https://www.ncbi.nlm.nih.gov/geo/query/acc.cgi?acc=GSE38557]
Sorted foetal liver cells RRBS GSE32214 [https://www.ncbi.nlm.nih.gov/geo/query/acc.cgi?acc=GSE32214]
Megakaryocyte embryo 14.5 ChIP-Seq (H3K4me1) ENCFF213NED [https://www.encodeproject.org/experiments/ENCSR000DHR/]
MEL ChIP-Seq (H3K4me3) ENCFF335CVP [https://www.encodeproject.org/experiments/ENCSR000CEX/]
MEL ChIP-Seq (H3K27ac) ENCFF352TNV [https://www.encodeproject.org/experiments/ENCSR000CEV/]
MEL DNase-Seq ENCFF155MQS [https://www.encodeproject.org/experiments/ENCSR446MUM/]
Megakaryocyte-erythroid progenitor cell ATAC-Seq ENCFF831CZO [https://www.encodeproject.org/experiments/ENCSR064IHX/]

Public available DNA methylation dynamics data link:
http://www.medical-epigenomics.org/papers/broad_mirror/invivomethylation/index.html

JASPAR database:
MA0035.2 (GATA1)

The source data underlying Figs 1b-d, 2e, 3c-d, 4a-d and 5c-d and Supplementary Figs 1a-b, 2a-b, 5, 7, 10a-d, 11b, 12a-c and 13 are provided as a Source Data file.

The data that support the findings of this study are available from the corresponding author upon reasonable request. Correspondence and requests for materials should be addressed to Merlin Crossley: m.crossley@unsw.edu.au.

Field-specific reporting

Please select the one below that is the best fit for your research. If you are not sure, read the appropriate sections before making your selection.

☒ Life sciences ☐ Behavioural & social sciences ☐ Ecological, evolutionary & environmental sciences

For a reference copy of the document with all sections, see nature.com/documents/nr-reporting-summary-flat.pdf

Life sciences study design

All studies must disclose on these points even when the disclosure is negative.

Sample size	Sample sizes were decided based on a combination of previous experimentation, pilot studies and power studies, with numbers selected as that necessary to observe phenotypic and genetic changes with statistical significance while reducing animal overconsumption.<br>For CRISPR/Ca9 genome edited cell lines, 4 independent clonal populations were used to validate the function of CGATA site at c-Kit intron 2. As control, 11 clonal wild type populations were used.<br>Three independent Tet2 knockout clonal populations were used to investigate the impact of DNA methylation on GATA-1 binding and regulation in G1E-ER4 cells.<br>4 wildtype C57BL/6J mice were used to check DNA methylation of target sites.<br>4 wild type mice and 4 homozygous c-Kit (C>T)GATA mutant mice (littermates) were used in flow cytometry analysis.
Data exclusions	No data was excluded
Replication	For the cell experiments, experimental results were able to be reproduced in different clonal populations.<br>For the mouse experiments, optimisation experiments were performed on mice prior to reaching backcross generation 5. For the c-Kit mouse line the experiment was performed on three separate occasions and data from only one is shown. The data from the other replicates was

	consistent with the data shown.
Randomization	Randomisation was not relevant to this study as mice were paired based on age, sex and litter.
Blinding	Investigators were not blinded to sample identity as all data produced was from objective quantitative methods so subjective bias was not relevant.

Reporting for specific materials, systems and methods

We require information from authors about some types of materials, experimental systems and methods used in many studies. Here, indicate whether each material, system or method listed is relevant to your study. If you are not sure if a list item applies to your research, read the appropriate section before selecting a response.

Materials & experimental systems

n/a	Involved in the study	
☐	☒	Antibodies
☐	☒	Eukaryotic cell lines
☒	☐	Palaeontology
☐	☒	Animals and other organisms
☒	☐	Human research participants
☒	☐	Clinical data

Methods

n/a	Involved in the study	
☒	☐	ChIP-seq
☐	☒	Flow cytometry
☒	☐	MRI-based neuroimaging

Antibodies

Antibodies used

GATA-1 antibody (Santa Cruz technology, #sc256),
Tet2 antibody (Abcam, #ab124297),
Actin antibody (Sigam, #a1978),

Lineage markers:
CD3 monoclonal antibody (145-2C11), biotin, eBioscience (ThermoFisher Scientific, #13-0037-82),
CD4 monoclonal anitbody (G1.5K), biotin, eBioscience (ThermoFisher Scientific, #13-0041-85),
CD11b monoclonal antibody (M1/70), biotin, eBioscience (ThermoFisher Scientific, #13-0112-85),
CD5 monoclonal antibody (53-7.3), biotin, eBioscience (ThermoFisher Scientific, #13-0051-85),
CD8 monoclonal antibody (53-6.7), biotin, eBioscience (ThermoFisher Scientific, #13-0081-85),
CD45R (B220) monoclonal antibody (RA3-6B2), biotin, eBioscience (ThermoFisher Scientific, #13-0452-85),
Ly-6G monoclonal antibody (RB6-8C5), biotin, eBioscience (ThermoFisher Scientific, #13-5931-85),
TER-119 monoclonal antibody (TER-119), biotin, eBioscience (ThermoFisher Scientific, #13-5921-82),
PE-CF594 Streptavidin (BD Bioscience, #562318),

other Flow cytometry fluorescent antibodies:
LY-6A/E(Sca1) antibody, V500 (BD Bioscience, #561229),
CD117 (cKit) antibody, APC-H7 (BD Bioscience, #560250),
CD34 antibody (RAM34), FITC (BD Bioscience, #553733),
CD16/32 antibody, PE-Cy7 (ThermoFisher, #25-0161-81),
DAPI (Life Technologies, #62248),
7-AAD (BIO-RAD, #1351102),
Ter119 monoclonal antibody, PE (BD Bioscience, #553673),
CD71 monoclonal antibody, FITC (Biolegend, #334104).

BrdU proliferation assay antibodies:
BrdU detection antibody (Cell Signaling Technology, #94079)
Anti-mouse IgG, HRP-linked antibody (Cell Signaling Technology, #34709)

Validation

All primary antibodies were validated by the suppliers.

Antibodies were validated for use in the system under study based on positive and negative controls. Positive and negative control loci were used to confirm the GATA-1 antibody was working for ChIP assays. COS-7 empty (negative) and transiently over-expressed proteins from COS-7 cells were used to confirm the GATA-1 antibody recognised the appropriate proteins in EMSA super-shifts. Wild type G1E-ER4 cells (positive) and Tet2 knockout G1E-ER4 cells were used to confirm the Tet2 antibody recognised the appropriate proteins in Western blots. Negative and positive cells/beads were used to confirm the fluorescent antibodies recognised specific cell markers for flow cytometry.

Eukaryotic cell lines

Policy information about cell lines

Cell line source(s)	G1E-ER4 cell line: gift from Professor Stu Orkin (Harvard Medical School Boston, USA)<br>CHO cell line: gift from Professor Stu Orkin (Harvard Medical School Boston, USA)<br>MEL cell line: gift from Professor Stu Orkin (Harvard Medical School Boston, USA)<br>COS-7 cell line: gift from Professor Stu Orkin (Harvard Medical School Boston, USA)
Authentication	None of the cell lines have been authenticated
Mycoplasma contamination	Cell lines were not tested for mycoplasma contamination
Commonly misidentified lines<br>(See ICLAC register)	No commonly misidentified cell lines were used

Animals and other organisms

Policy information about studies involving animals; ARRIVE guidelines recommended for reporting animal research

Laboratory animals	C57BL/6J mice, 8-12 weeks old, male and female.<br>Swiss mice, 8-12 weeks old, female.<br>Appropriate Housing conditions:<br>Temperature: 22C (+/-2C)<br>Humidity: 55% (+/-10%)
Wild animals	The study did not involve wild animals
Field-collected samples	The study did not involve samples collected from the field
Ethics oversight	All animal work was carried out in accordance with approval from the UNSW Animal Care and Ethics Committee (Approval Nos. 16/5B and 18/156B).

Note that full information on the approval of the study protocol must also be provided in the manuscript.

Flow Cytometry

Plots

Confirm that:

- [x] The axis labels state the marker and fluorochrome used (e.g. CD4-FITC).
- [x] The axis scales are clearly visible. Include numbers along axes only for bottom left plot of group (a 'group' is an analysis of identical markers).
- [ ] All plots are contour plots with outliers or pseudocolor plots.
- [x] A numerical value for number of cells or percentage (with statistics) is provided.

Methodology

Sample preparation	For mouse haematopoietic stem cell and progenitor flow cytometry:<br>Mouse bone marrow was lysed with 9ml RO water for 10 seconds followed by adding 1ml 10xPBS to get rid of mature red blood cells. Lysed bone marrow cells were stained with biotin-conjugated Lineage marker antibody cocktail (CD3e, CD4, CD5, CD8a, CD45R/B220, Gr1, CD11b, Ter119) at 4 degree for 30 minutes. Stained cells were washed with FACs buffer (1xPBS, 5% FCS, 2mM EDTA) and centrifuged at 300g at 4 degree for 5 minutes. Cells were resuspended with FACs buffer and stained with PE-CF594 streptavidin, V500-conjugated Sca1, APC H7-conjugated c-Kit, PE-Cy7-conjugated CD16/32, FITC-conjugated CD34 and DAPI at 4 degree for 30 minutes. After full staining, cells were washed with 3ml FACs buffer and centrifuged at 300g at 4 degree for 5 minutes.<br><br>For mouse nulceated erythrocytes flow cytometry:<br>Mouse bone marrow was stained with FITC-conjugated CD71, PE-conjugated Ter119 and DAPI at 4 degree for 30 minutes. Stained cells were washed with 3ml FACs buffer (1xPBS, 5% FCS, 2mM EDTA) and centrifuged at 300g at 4 degree for 5 minutes. Cells were resuspended with FACs buffer.<br><br>For CRISPR/Cas9 cell sorting:<br>Cells containing Cas9, eGFP and sgRNA were stained with 7-AAD at 4 degree for 30 minutes. Stained cells were washed with FACs buffer and centrifuged at 300g at 4 degree for 5 minutes. Cells were resuspended with FACs buffer.
Instrument	BD LSRFortessa SORP flow cytometer and BD FACSAria II cell sorter

Software	BD FACSDiva software and FlowJo software
Cell population abundance	For mouse haematopoietic stem cell and progenitor flow cytometry:<br>We recorded approximately 10 million events for each mouse bone marrow samples. After sorting out live and single cells, almost 5 million cells were left per sample. We had 2-30,000 cells that were lineage negative, Sca1 negative and cKit positive populations that, in turn, were further sorted into megakaryocyte-erythroid progenitors (MEP), common-myeloid progenitors (CMP) and granulocyte-monocyte progenitors (GMP).<br><br>For mouse nucleated erythrocytes flow cytometry:<br>We recorded approximately 4 million events for each mouse bone marrow samples. After sorting out live and single cells, almost 3 million cells were left per sample. We had around 30,000 cells that were CD71 positive, Ter119 positive and DAPI negative populations.
Gating strategy	For mouse haematopoietic stem cell and progenitor flow cytometry:<br>1) Gate all blood cells with FSC-A and SSC-A channels; 2) Gate all single cells with SSC-A and SSC-H channels; 3) Gate all live cells with SSC-A and DAPI channels; 4) Gate Lineage negative cells with SSC-A and Lineage-PE-CF594 channels; 5) Gate cKit positive, Sca1 negative cells with Scal1-V500 and cKit-APC-H7 channels; 6) Gate cells with different expression level of CD34 and CD16/32 cell surface markers with CD34-FITC and CD16/32-PE-Cy7 channels: CD34 negative and CD16/32 low are megakaryocyte-erythroid progenitors, CD34 positive and CD16/32 medium are common-myeloid progentiors, CD34 positive and CD16/32 high are granulocyte-monocyte progenitors.<br><br>For For mouse nucleated erythrocytes flow cytometry:<br>1) Gate all blood cells with FSC-A and SSC-A channels; 2) Gate all single cells with SSC-A and SSC-H channels; 3) Gate all live cells with SSC-A and DAPI channels; 4) Gate cells with CD7-FITC and Ter119-PE channels. CD71-FITC positive and Ter119-PE positive are nucleated erythrocytes. CD71 low Ter119 low are non-erythroid cells. CD71 high Ter119 medium cells are proerythroblasts. CD71 high Ter119 high cells are basophilic erythroblasts. CD71 medium Ter119 high cells are polychromatophilic erythroblasts. CD71 low Ter119 high cells are more mature orthochromatophilic erythroblasts and reticulocytes.<br><br>For CRISPR/Cas9 cell sorting:<br>1)Gate all cells with FSC-A and SSC-A channels; 2) Gate all single cells with SSC-A and SSC-H channels; 3) Gate all live cells with SSC-A and 7-AAD channels; 4) Gate and sort cells with GFP positive signal.

☒ Tick this box to confirm that a figure exemplifying the gating strategy is provided in the Supplementary Information.
